# Gamma irradiation as a grape pomace stabilisation treatment: Assessment of phenolic and mineral preservation

**DOI:** 10.1016/j.fochx.2026.104435

**Published:** 2026-09-08

**Authors:** María Dolores López-Belchí, Daniel Villegas, Raúl Domínguez-Perles, Sonia Medina, Cristina García-Viguera, Bárbara Hormazabal, V. Felipe Laurie

**Affiliations:** a Departamento de Producción Vegetal, Facultad de Agronomía, Universidad de Concepción, 4030000 Concepción, Chile; b Laboratorio de Bioactivos e Ingredientes Vegetales (BIOINVE), Centro de Biotecnología, Universidad de Concepción, 4030000 Concepción, Chile; c Laboratorio de Radiobiología Vegetal, Comisión Chilena de Energía Nuclear; Santiago, Chile; d Lab. Fitoquímica y Alimentos saludables (LabFAS). Dept. of Food Science and Technology, CSIC, CEBAS, Campus Universitario de Espinardo 25, 30100 Murcia, Spain; e Departamento de Horticultura, Facultad de Ciencias Agrarias, Universidad de Talca, Chile; f Centro de Investigación e Innovación VitiScience -CIA, 250013, Santiago, Chile

**Keywords:** By-product valorisation, Non-thermal stabilisation, Phenolic compounds, Mineral profile, Wine

## Abstract

Grape pomace is a winery by-product whose valorisation requires stabilisation strategies to preserve quality during storage. This study assessed gamma irradiation (gamma-IR) as a non-thermal treatment for two commercial grape pomace matrices, unfermented ‘Sauvignon blanc’ (SBP) and fermented ‘Malbec’ pomaces. Samples were exposed to 0–2000 Gy and analysed after 1 and 30 days for phenolic stability, mineral composition, and mineral–phenolic relationships. Effects depended on dose, matrix, and storage. Doses of 500–1000 Gy preserved phenolics with minor mineral changes, whereas higher doses increased phenolic losses. At day 30, non-coloured phenolics in SBP varied by less than 16% from day-1 levels at 500–1000 Gy but decreased by 31% at 2000 Gy. DPPH antioxidant capacity declined by 39–52% in SBP and 8–49% in Malbec pomace. In SBP, the initial bacterial reduction was not sustained after 30 days of refrigerated storage. Exploratory correlations suggested mineral–phenolic associations. Overall, gamma-IR shows promise as a matrix-specific pretreatment.

## Highlights

• Gamma-IR at 500–1000 Gy preserved phenolics and minerals in pomace.
• White and red grape pomaces responded differently to gamma irradiation.
• Gamma-IR modulated phenolics while minerals remained mostly stable.
• Phenols and minerals contributed to treatment-related multivariate patterns.
• Gamma-IR showed matrix-dependent potential for pomace stabilisation.

## Graphical abstract

Assessment of Gamma Irradiation as Grape Pomace Stabilisation Treatment for Phenolic and Mineral Preservation.

**Figure:**
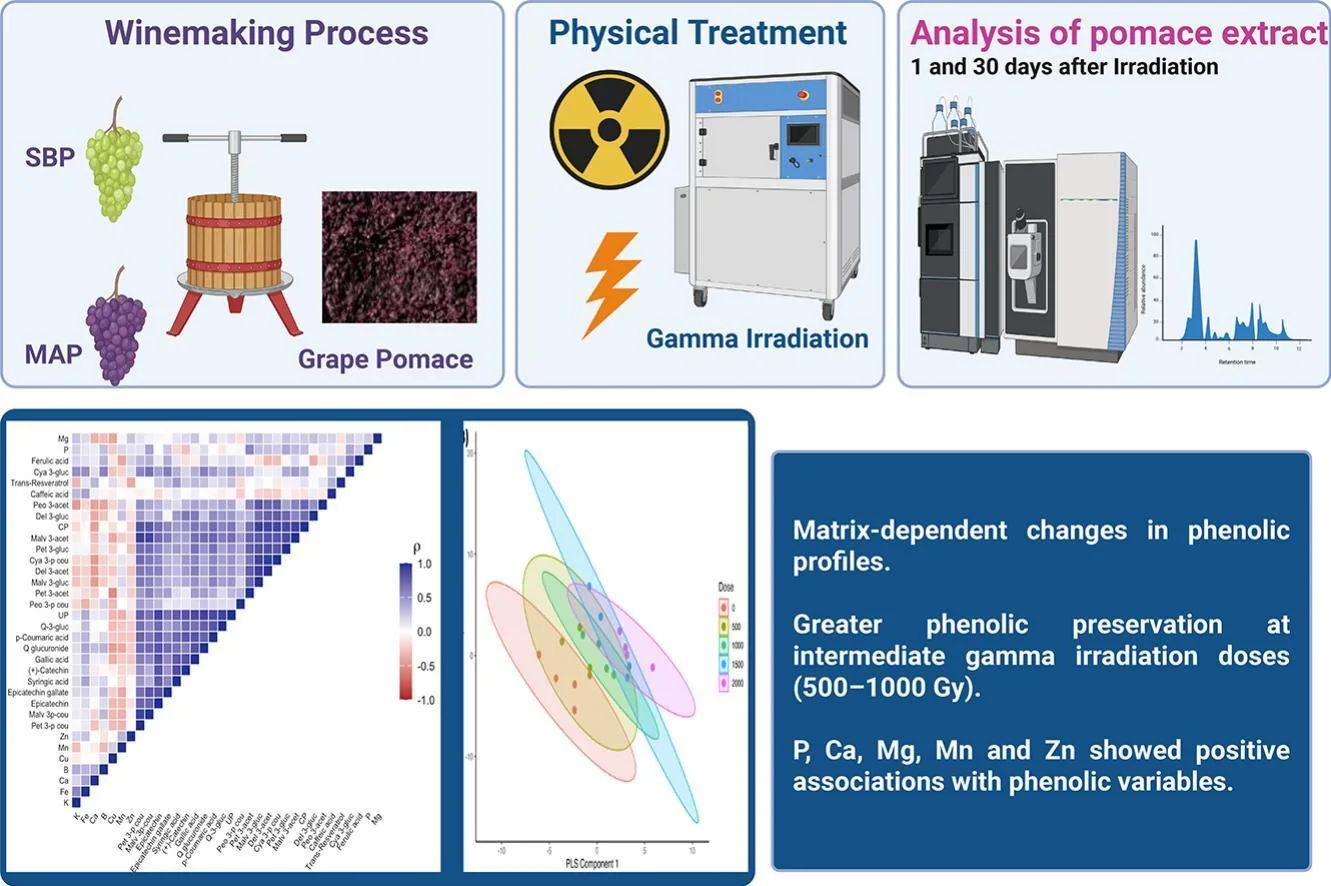

## 1 Introduction

Grape (*Vitis vinifera* L.) pomace is a solid by-product remaining from grapes after juice extraction or fermentation, which makes up to 30% of the total weight of grapes processed (He et al., 2020), containing skins, seeds, and (occasionally) stems. These grape tissues have been promoted as valuable sources of dietary fibre, (poly)phenols, and oils (Caponio et al., 2023; Onache et al., 2022; Vinha et al., 2023). Despite the interest of these grape pomace constituents for the industries of food and feed, cosmetics, agriculture, chemistry, and materials/energy (Caponio et al., 2023; Olate-Olave et al., 2025), this by-product is frequently discarded, thereby losing the opportunity for valorisation.

The (poly)phenolic content of grape pomace varies significantly between red and white grapes, not only due to their intrinsic composition (Teixeira et al., 2014), but also because red winemaking involves the co-fermentation of grape must and pomace. In contrast, to obtain white wine, musts are fermented separately. These contrasting processing conditions contribute to differences in the phenolic compositions of the resulting pomaces. According to these technical processes, grape pomace obtained during the manufacture of red wines is particularly rich in anthocyanins (polyphenols responsible for the red colour and antioxidant properties). Alternatively, the unfermented pomace obtained after pressing white grapes have higher concentrations of flavonols and proanthocyanidins (Capakova et al., 2018; López-Belchí et al., 2021; Onache et al., 2022). Therefore, red and white grape pomaces should be considered as contrasting commercial matrices shaped by both cultivar and winemaking-related processing conditions.

Recent advances in food processing technologies have been applied to grape pomace to enhance its bioactive compound profile and shelf life (López-Belchí et al., 2021). Among all, gamma-IR minimises the degradation of heat-sensitive compounds compared with traditional thermal processing methods (Jeong et al., 2024). This may be particularly significant for preservation of heat-sensitive constituents is desired.

The broadly reported biological benefits of grape pomace phenolic compounds include anti-inflammatory and cardioprotective effects (Balea et al., 2018; Sánchez-Bravo et al., 2025; Vinha et al., 2023). However, it is worth mentioning that the quantitative profile of bioactive (poly)phenols and the associated tentative health benefits may vary depending on the grape variety and processing method (González-Centeno et al., 2013; Guaita & Bosso, 2019; Xu et al., 2016). Low-to-moderate gamma irradiation doses have been applied in food matrices for microbial control. For example, doses of 1–5 kGy significantly reduced the growth of *Fusarium moniliforme* in contaminated corn (Mansur et al., 2014). However, information regarding the effects of comparable doses on the phenolic composition of grape pomace remains limited. Previous studies have reported irradiation-related changes in the antioxidant activity of grape pomace (Yoon et al., 2010) and have evaluated combined preservation treatments including gamma irradiation during low-temperature storage (Augustine et al., 2013).

Beyond (poly)phenols, grape pomace has also been demonstrated as a source of mineral nutrients and trace elements, whose concentration could be altered by microorganisms during processing and/or storage (Fernandes et al., 2025; Prata et al., 2025; Sagdic et al., 2014). In this regard, gamma-IR is expected to target microbial contaminants without significantly affecting the inherent mineral profile.

In addition, mineral elements may also influence compositional stability by participating in oxidative or redox-related reactions and by interacting with phenolics through complexation or chelation mechanisms. Grape pomace contains minerals such as potassium (*K*), calcium (*Ca*), magnesium (*Mg*), sodium (*Na*), phosphorus (*P*), and iron (*Fe*), which are associated with nutritional and physiological relevance (Sagdic et al., 2014). Given the chemical stability of minerals, gamma-IR is not expected to modify the elemental composition itself; however, it may influence the recoverable mineral content by altering matrix structure, solubility, or extractability. In analogous plant-derived matrices, gamma irradiation has been investigated as a preservation strategy for extending shelf life while maintaining nutritional quality under appropriate treatment conditions (Shami & Naz, 2019). However, its influence on recoverable mineral concentrations in grape pomace remains poorly documented.

Therefore, this study aimed to evaluate the effect of low-to-moderate gamma irradiation doses on the phenolic stability and mineral composition of two contrasting commercial grape pomace matrices, unfermented Sauvignon blanc pomace and fermented Malbec pomace. Samples were analysed after 1 and 30 days of storage to assess dose-, matrix-, and storage-dependent responses. In addition, mineral–phenolic associations were explored to provide an integrated interpretation of grape pomace compositional stability after irradiation.

## 2 Materials and methods

### 2.1 Chemical reagents

Phenolic standards as well as Trolox, fluorescein (free acid), 2,2′-azobis(2-methylpropanimidine) dihydrochloride (AAPH), 2,2-diphenyl-1-picrylhydrazyl (DPPH), methylcellulose, and ammonium sulfate were acquired from Sigma-Aldrich (St. Louis, MO, USA). All chromatographic solvents (acetonitrile, methanol, ethyl acetate, 2-butanone, and formic acid) were of analytical grade and acquired from Merck (Darmstadt, Germany).

### 2.2 Experimental design: Sampling and irradiation treatments

This study was performed with grape (*Vitis vinifera* L.) pomace of two contrasting commercial matrices produced in the Maule Region (Chile), unfermented ‘Sauvignon blanc’ (SBP) from Hualañe (Curicó, Chile) and fermented ‘Malbec’ (MAP) from Cauquenes (Cauquenes, Chile). Both of them obtained from “Viña Santa Carolina” during the 2024 harvest. ‘Sauvignon blanc’, the white variety of grapes were mechanically destemmed and crushed, and skins were pressed following standard winery protocols. Once generated, 20 kg of grape pomace were sampled. To obtain red grape pomace (‘Malbec’), grapes were fermented and racked using an AirMixing MI™ system and 20 kg of pomace were sourced. Once sampled, both pomace varieties were immediately frozen at −80 °C. For each 20 kg bulk sample, four 1 kg subsamples were randomly withdrawn and placed in separate bags. For each irradiation dose (0, 500, 1000, 1500, and 2000 Gy), a 200 g sample was collected from each source bag and divided, prior to irradiation, into two separate 100 g experimental units, one assigned to day 1 and the other to day 30 of post-irradiation storage. Each experimental unit was irradiated and stored independently. Accordingly, each combination of irradiation dose and storage time comprised four experimental units (*n* = 4), each originating from a different source bag. The source bag was included as the blocking factor. Since separate experimental units were prepared for each storage time, samples analysed on days 1 and 30 represented independent destructive units rather than repeated measurements. Following irradiation, samples were stored as non-dried grape pomace at 4 °C in capped polypropylene tubes with atmospheric headspace and protected from light until analysis. Moisture content and water activity were not monitored during storage and should therefore be considered when interpreting microbial recovery and phenolic stability. These two materials corresponded to two specific commercial bulk lots obtained under standard winemaking conditions. The aim was not to isolate the effect of fermentation itself, but to evaluate the response of these two contrasting grape pomace lots to gamma irradiation. Accordingly, differences between SBP and MAP were interpreted as differences between the specific matrices evaluated rather than as effects attributable solely to cultivar or fermentation. Because one commercial bulk lot was evaluated for each pomace matrix, inference at the matrix level is restricted to these materials.

Irradiation treatments were conducted at the Irradiation Laboratory of the Chilean Nuclear Energy Commission. A self-shielded irradiator (GammaCell 220R, JL Shepherd, San Fernando, CA, USA) equipped with 21 cobalt-60 (60Co) pencils arranged radially, with an approximate activity of 500 Ci, was used. The irradiation chamber was dosimetrically characterised following the Fricke reference method as specified in ASTM E-1026 (ASTM International, 2023). The established dose rate of 5.44 Gy/min was used to calculate the irradiation times for the treatments. Fricke dosimeters positioned at different locations within the irradiation volume indicated spatial dose-rate variation below 3%; however, the dose uniformity ratio across the sample volume and the combined absorbed-dose uncertainty were not independently determined. Samples were transported under chilled conditions and irradiated in a non-frozen state at approximately 18 °C. Each 100 g experimental unit was distributed among capped 50-mL polypropylene tubes, which were irradiated simultaneously under atmospheric air and positioned at the same height within the irradiation chamber. The selected dose range of 0.5–2.0 kGy was intended to evaluate a relatively mild treatment rather than sterilisation. The experiment was arranged as a randomised complete block design, with the four source bags serving as blocks.

### 2.3 Microbiological characterisation of grape pomace

The microbiological profile (bacteria and fungi) was analysed, and the results are summarised as supporting information (Table S1). Sterile saline solution (0.89% NaCl, *w*/*v*) was prepared by dissolving NaCl in deionised water, followed by sterilisation by autoclaving at 121 °C for 15 min. After cooling to room temperature, the solution was handled under aseptic conditions. From each experimental unit, 10 g of homogenised fresh grape pomace was suspended in 100 mL of sterile saline solution in an Erlenmeyer flask and shaken at 150 rpm for 2 h. Ten-fold serial dilutions were prepared up to 10^−5^. Aliquots from dilutions 10^−1^ to 10^−5^ were plated on Plate Count Agar (PCA). Culture media were prepared in deionised water, autoclaved at 120 °C for 20 min, and then poured (10 mL per dish) under aseptic conditions in a laminar flow hood. For microbial counts, 100 μL of each dilution was spread onto the agar surface and incubated at 25 ± 2 °C in the dark for 24 h. Colony-forming units (CFU) were quantified on plates, and the results were expressed as log_10_ CFU/g of fresh grape pomace weight (fw). Fungal communities were evaluated using Sabouraud Dextrose Agar (SDA), a medium selective for yeasts and filamentous fungi. Briefly, 10 g from each experimental unit was processed as described for bacterial quantification and plated on SDA. Plates were incubated at 25 °C for 3–5 days, and afterwards, fungal colonies were counted (expressed as log_10_ CFU/g fw) (Pitt & Hocking, 2009). Based on the lowest dilution plated (10^−1^) and the inoculated volume (100 μL), the limit of detection for both bacterial and fungal counts was 1.0 × 10^3^ CFU/g fw, equivalent to 3.00 log₁₀ CFU/g fw. Values below this limit were reported as <LOD. Four experimental units, one originating from each source bag, were analysed for each combination of irradiation dose and storage time (*n* = 4).

### 2.4 Mineral content

For assessing the content of mineral nutrients and trace elements, samples were frozen at −80 °C and then freeze-dried (Lyophilizer OPERON, Korea). The mineral content in grape pomace was determined following a dry ashing method (Thiex et al., 2012). Briefly, the dried samples were incinerated in a muffle furnace at 500 °C for 6 h to obtain ash. The resulting ash was dissolved in HCl and filtered to obtain the mineral extract. Mineral nutrients, including phosphorus (P), potassium (K), calcium (Ca), and magnesium (Mg), and trace elements, including iron (Fe), manganese (Mn), zinc (Zn), copper (Cu), and boron (B), were quantified in four experimental units per treatment combination (*n* = 4) using inductively coupled plasma optical emission spectrometry (ICP-OES, Optima 8000, PerkinElmer, Waltham, MA, USA). Calibration was performed using certified multielement standards prepared in 1% HCl. The method LOQs were 5.0, 9.0, 6.1, 5.3, 27.5, 11.0, 3.5, 3.5, and 8.1 μg/g dw for P, K, Ca, Mg, Fe, Mn, Zn, Cu, and B. Mineral concentrations were expressed as μg/g dw.

### 2.5 (poly)phenolic extraction and HPLC-DAD-ESI-MSn quantitative profiling of grape pomace from ‘sauvignon blanc’ and ‘malbec’

To extract phenolic compounds, freeze-dried material (500 mg) from each experimental unit were extracted with 5 mL of an extracting solvent consisting of methanol/deionised water/formic acid (25:24:1, *v/v/v*) (Costa-Pérez et al., 2023). This hydroalcoholic acidified solvent was selected because methanol-water provides suitable polarity for extracting a broad range of phenolic compounds, while formic acid contributes to the stabilisation of acid-sensitive phenolics, particularly anthocyanins, during extraction. The mixture was stirred for 5 min, sonicated for 1 h, and kept at 4 °C overnight. Then, samples were centrifuged at 10000 rpm for 10 min and filtered through 0.22 μm PVDF syringe membranes (Millex V13, Millipore, Bedford, MA, USA). The extracts were kept at 4 °C until spectrophotometric and chromatographic analyses.

Phenolic analyses were carried out by using an Agilent HPLC 1100 series system, comprising a photodiode array detector and a mass detector in tandem (Agilent Technologies, Waldbronn, Germany). The setup featured a binary pump (model G1312A), an autosampler (model G1313A), a degasser (model G1322A), and a photodiode array detector (model G1315B), controlled with the ChemStation software (Agilent, version 08.03). The chromatographic separation was carried out using a C18 Luna column (Phenomenex, Torrens, CA, USA) of 250 × 4.6 mm length and diameter, 5 μm and 100 Å pore size, and Security Guard Cartridges PFD C18 (4 × 3.0 mm). The mobile phases consisted of deionised water/formic acid (99.0:1.0, *v/v*) (phase A) and methanol (phase B). The flow rate and injection volume were 0.9 mL/min and 20 μL, respectively. The mass detector was an ion trap spectrometer (model G2445A) equipped with an ESI interface, and operated *via* the LCMSD software (Agilent, version 4.1). Ionisation conditions were optimised with a capillary temperature of 350 °C and 4 kV voltage. The nebuliser pressure was set to 65.0 psi, with a nitrogen flow rate of 11 L/min. Full-scan mass spectrometry was applied, covering the *m/z* range from 100 to 1200. The collision-induced fragmentation experiments in the ion trap employed helium as the collision gas, with voltage ramping from 0.3 to 2 V. Spectral data were collected in negative (proanthocyanidins and catechin derivatives, phenolic acids, stilbenes, and flavonols) and positive (anthocyanins) ionisation modes. MSn experiments were performed automatically on the most abundant fragment ions derived from MS(n − 1). Calibration curves were freshly prepared each day of analysis using authentic standards (catechin, *p*-coumaric acid, *trans*-resveratrol, quercetin-3-*O*-rutinoside, and malvidin-3-*O*-glucoside) and quantified at 280, 320, 320, 360, and 520 nm, respectively. Calibration ranges were established according to the linear response of the analytical method. LOD and LOQ were determined from the chromatographic signal-to-noise ratio and expressed in the same final units used for reporting sample concentrations. LOD, limit of detection; LOQ, limit of quantification (Table S2). Each experimental unit was extracted and analysed independently, resulting in four experimental replicates per treatment combination (*n* = 4). No separate repeatability or matrix-spike recovery assessment was performed as part of this study. Some phenolic derivatives were quantified as equivalents of representative class standards, as indicated in Table S2. The results are expressed as μg/g dw.

The total content of non-coloured and coloured phenolic compounds were calculated as the sum of individual compounds quantified by LC-MS and expressed as μg/g dw.

### 2.6 Antioxidant capacity of grape pomace from ‘sauvignon blanc’ and ‘malbec’

The assays of 2,2-diphenyl-1-picrylhydrazyl (DPPH) radical scavenging (Mena et al., 2011) and the oxygen radical absorbance capacity (ORAC_FL_) (Romero-Román et al., 2021) were used to measure the antioxidant activity of the white and red grape pomaces*.* The DPPH• radical scavenging activity was determined from the change in absorbance at 515 nm after 30 min of incubation. Antioxidant capacity by ORACFL was evaluated from the fluorescence variation of grape pomace samples after 120 min of reaction with the radical species. Analyses were performed in 96-well flat-bottom microplates, using black microplates for ORACFL, with a Synergy H1 hybrid multi-mode microplate reader (Biotek, Winooski, VT, USA). Results were expressed as μg Trolox equivalents per gram of dry weight (μg TE/g dw). Four experimental units were analysed for each combination of irradiation dose and storage time (*n* = 4).

### 2.7 Statistical analyses

Results were expressed as mean ± standard deviation (SD) of four experimental units (*n* = 4). Model residuals were assessed for normality using the Shapiro–Wilk test and Q–Q plots, whereas homogeneity of variances was evaluated using Levene's test. For each pomace matrix, the effects of gamma-IR dose, storage time, and their interaction were analysed using a two-way ANOVA within a randomised complete block design, with source bag included as the blocking factor. In addition, a three-way ANOVA was performed to evaluate the effects of pomace matrix, gamma-IR dose, storage time, and their interactions, with source bag nested within pomace matrix included as the blocking factor. Since separate experimental units were prepared for days 1 and 30 before irradiation, storage time was not treated as a repeated-measures factor. For the matrix-specific compound-wise ANOVAs reported in Tables S3 and S4, raw *p*-values were adjusted using the Benjamini–Hochberg false discovery rate procedure separately for each pomace matrix and model term. Significant effects were followed by Tukey-adjusted comparisons of estimated marginal means, including within-factor comparisons for significant interactions. Significance was set at q < 0.05 for ANOVA effects and *p* < 0.05 for pairwise contrasts. Analyses were performed using R software (v. 4.1.0).

Spearman correlation analysis was performed separately for each pomace matrix and storage time using observations pooled across the five irradiation doses (*n* = 20; five doses × four experimental blocks) to assess exploratory associations among phenolic, mineral, and antioxidant variables. Partial Least Squares Discriminant Analysis (PLS-DA) was applied as an exploratory supervised approach to visualise treatment-related patterns in the combined phenolic, mineral, and antioxidant datasets. Separate models were fitted for each pomace matrix and storage time using two latent components. Before model fitting, variables were centred and scaled to unit variance. Model performance was evaluated using four-fold leave-one-source-bag-out cross-validation, in which all five irradiation treatments originating from the same source bag were assigned to the same validation fold. The proportion of variance explained in the predictor and response matrices (R^2^X and R^2^Y, respectively), cross-validated predictive ability (Q^2^), classification accuracy, and error rate were calculated. Model significance was further assessed using 999 permutations of the irradiation-dose labels within each source bag, thereby preserving the blocked experimental structure. Variable Importance in Projection (VIP) scores were calculated, and variables with VIP scores >1 were considered candidate contributors to treatment separation rather than validated biomarkers. All multivariate analyses were performed in *R* using the FactoMineR, mixOmics, corrplot, PLS, and ggplot2 packages.

## 3 Results and discussion

### 3.1 Microbiological characterisation of grape pomace after gamma-IR

Gamma-IR has been widely applied to control microbiological contamination in food matrices, with well-documented benefits for food safety and shelf life (Monk et al., 1995; Smith & Pillai, 2004). Although microbial control was not the primary objective of this study, bacterial and fungal loads were analysed to verify the antimicrobial effectiveness of gamma-IR treatments and to provide complementary information on microbial load after treatment (Table S1).

At day 1, ‘Sauvignon blanc’ pomace (SBP) exhibited higher bacterial counts than ‘Malbec’ pomace (MAP), likely due to the absence of antimicrobial conditions associated with red wine fermentation, such as exposure to ethanol. Accordingly, irradiation controls (0 Gy) showed a bacterial load of 7.52 ± 0.17 log_10_ CFU/g fw in SBP, whereas bacterial counts in MAP were below the limit of detection at the same time point (Table S1). In SBP, gamma-IR induced a dose-dependent reduction in bacterial load at day 1, reaching 4.49 ± 0.31 log_10_ CFU/g at 2000 Gy. However, bacterial recovery was observed after 30 days of storage, even at the highest dose. The present analyses do not allow us to distinguish between survival and subsequent growth of irradiation-resistant microorganisms and post-treatment recontamination (De Bock et al., 2023).

In contrast, bacterial counts in MAP remained below the limit of detection at both day 1 or day 30 across all irradiation doses, including the non-irradiated control. In addition, fungal counts remained below the limit of detection in all analysed samples at both time points, indicating a low initial fungal load (Dril, 2021).

These results indicated that gamma-IR was an effective tool for reducing bacterial load, specifically in SBP shortly after treatment. However, bacterial recovery during storage showed that gamma-IR alone did not ensure the microbiological stabilisation of SBP over 30 days at 4 °C. Since bacterial counts in MAP and fungal counts in both pomace matrices were below the limit of detection even in the non-irradiated controls, these findings cannot be attributed specifically to gamma-IR, and it would be necessary to increase the dose of gamma-IR or combine the treatment with other preservation strategies (*e.g.*, high hydrostatic pressure, pulse electric fields, or ultrasound processing, among other) (Salar et al., 2023) to prevent microbial proliferation during storage.

### 3.2 Mineral content of grape pomace after gamma-IR treatments

The main macronutrients quantified in grape pomace from SBP and MAP showed stability throughout the experiment, despite the presence of statistically significant effects associated with gamma-IR dose, pomace type, and post-irradiation storage time (Table 1) (Goulas et al., 2021; Xu et al., 2016).

**Table 1:** Mineral nutrient composition (μg/g dw) of ‘Sauvignon blanc’ and ‘Malbec’ pomace (SBP and MAP, respectively) after gamma irradiation and storage.

Pomace matrix	Irradiation dose (Gy)	P		K		Ca		Mg	
		Day 1	Day 30	Day 1	Day 30	Day 1	Day 30	Day 1	Day 30
SBP	0	1317.7 ± 4.5	1446.0 ± 41.5	7505.2 ± 363.3	5585.2 ± 1541.4	1656.7 ± 95.8	2055.0 ± 55.6	632.2 ± 37.3	697.5 ± 62.3
SBP	500	1392.7 ± 39.7	1483.5 ± 71.6	6494.2 ± 169.4	5668.5 ± 37.8	2092.5 ± 196.4	2338.5 ± 49.5	675.0 ± 62.8	697.5 ± 75.0
SBP	1000	1490.0 ± 22.3	1350.0 ± 123.0	6250.5 ± 281.8	5364.7 ± 146.6	2144.2 ± 280.0	1780.0 ± 28.3	720.0 ± 27.4	585.7 ± 29.5
SBP	1500	1486.5 ± 12.6	1443.0 ± 62.7	5712.7 ± 53.7	6541.5 ± 506.2	1989.0 ± 122.8	2208.0 ± 138.7	718.5 ± 130.5	729.7 ± 141.3
SBP	2000	1455.0 ± 4.2	1548.0 ± 28.8	4275.0 ± 157.8	5321.2 ± 162.6	2022.0 ± 263.3	2378.2 ± 138.7	747.0 ± 8.8	821.2 ± 27.1
MAP	0	1331.2 ± 11.6	1317.7 ± 5.7	10,927.5 ± 353.6	12,386.8 ± 165.0	2791.5 ± 47.0	2843.5 ± 199.0	572.2 ± 29.7	554.2 ± 65.4
MAP	500	1308.0 ± 4.2	1384.5 ± 21.1	11,936.2 ± 385.1	10,787.2 ± 454.2	2772.7 ± 108.9	2710.7 ± 40.8	528.7 ± 15.4	528.7 ± 65.0
MAP	1000	1146.7 ± 4.0	1405.5 ± 15.2	10,717.5 ± 375.8	11,567.5 ± 459.2	2832.0 ± 45.5	2754.5 ± 132.2	471.0 ± 18.2	597.7 ± 41.5
MAP	1500	1245.7 ± 69.1	1192.5 ± 38.1	9429.7 ± 317.2	10,645.5 ± 751.8	2922.0 ± 247.6	2698.5 ± 9.3	540.0 ± 64.8	519.0 ± 66.7
MAP	2000	1391.2 ± 94.7	1275.0 ± 48.1	9477.7 ± 755.1	10,737.0 ± 183.1	2773.2 ± 119.4	2765.2 ± 77.9	674.2 ± 187.9	518.2 ± 71.7
Model		***		***		***		***	
Irradiation dose (I)		***		***		**		**	
Pomace matrix (M)		***		***		***		***	
Storage time (D)		*		n.s.		n.s.		n.s.	
I × M		***		***		***		n.s.	
I × D		**		***		***		n.s.	
M × D		n.s.		***		***		n.s.	
I × M × D		***		***		**		***	

Values are expressed as mean ± SD (*n* = 4). SBP, Sauvignon blanc pomace; MAP, Malbec pomace; dw, dry weight. Data were subjected to three-way ANOVA within a randomised complete block design, with irradiation dose (I), pomace matrix (M), and storage time after irradiation (D) as fixed factors. Significance symbols refer to raw *p*-values from the three-way ANOVA: n.s., not significant; *, *p* < 0.05; **, *p* < 0.01; ***, *p* < 0.001.

Three-way ANOVA revealed significant interactions between irradiation dose (I), pomace type (P), and storage time (D) for *P* and *K*, indicating that the response to gamma-IR was dependent on both the pomace matrix and the time elapsed after treatment. In SBP, phosphorus content increased at moderate irradiation doses shortly after treatment (day 1), whereas these differences were stabilised after 30 days of storage. In contrast, MAP showed less consistent changes in *P*, although storage time still influenced its response to irradiation. *Ca* and *Mg* also exhibited interaction effects involving pomace type and storage time, reflecting matrix-dependent behaviour following irradiation (Table 1).

Analysing the individual effects, *K* was the mineral most clearly affected by gamma-IR. In both pomaces, *K* content progressively decreased as irradiation dose increased, with lower values generally observed at day 30 compared to day 1, particularly up to 1000 Gy. This pattern was more evident in SBP, suggesting a higher sensitivity of this unfermented matrix to irradiation and subsequent storage. In contrast, *Ca* content remained relatively stable across doses and storage times, especially in MAP (Table 1).

*Mg* and *P* showed more moderate and variable responses. In SBP, higher recoverable concentrations of *Mg* and *P* contents were observed at higher doses (1500–2000 Gy), particularly after 30 days of storage, whereas MAP displayed only minor fluctuations without a clear dose-dependent pattern. Hence, MAP appeared less responsive to irradiation than SBP, consistent with the lower magnitude of changes observed across all minerals evaluated (Table 1).

These results indicate that gamma-IR induces only minor modifications in the recoverable macronutrient composition of grape pomace, with potassium being the most sensitive element to increasing irradiation doses and storage time. The more pronounced effects observed in SBP compared to MAP highlight the importance of pomace matrix characteristics, likely associated with differences arising from fermentation processes (Tsoupras et al., 2023).

These observed variations in mineral content may be associated with several non-exclusive mechanisms. Gamma-IR can induce structural modifications in plant tissues, potentially promoting mineral redistribution between insoluble and soluble fractions and facilitating their recovery during acid digestion. In addition, irradiation-related dry matter losses could result in an apparent concentration of remaining elements, while oxidative reactions may alter the chemical state of certain minerals, affecting their solubility. For highly soluble elements such as potassium, irradiation-induced tissue degradation combined with storage conditions may favour leaching processes, leading to the decreases observed, particularly at higher doses. Previous studies have reported that mineral composition in grape-derived matrices is strongly influenced by cultivar, growing conditions, winemaking practices, and processing background (Caponio et al., 2023; Kunová et al., 2019; Silva et al., 2021). García-Santos et al. described high concentrations of *K*, *Ca*, *Mg*, and *P* in grape pomace from ‘BRS Vitória’, highlighting the role of cultivar-related factors. Similarly, Rico et al. (2024) reported varietal differences in *K* response following irradiation treatments, underscoring the relevance of matrix-dependent effects. In this context, the present study provides evidence that moderate gamma-IR doses (500–1000 Gy) were associated with changes in the recoverable concentrations of some minerals, including P, Ca, and Mg, although these responses depended on pomace matrix and storage time (Garcia-Santos et al., 2025).

Grape pomace-derived materials have been explored as ingredients for functional beverages (Gerardi et al., 2020). This emphasises the need for in-depth exploration of the detailed mechanisms, especially concerning higher doses of irradiation that may change mineral content.

Variations in trace element concentrations were observed between grape pomace types following gamma-IR (Table 2). The statistical analysis revealed significant effects of irradiation dose (I), pomace matrix (M), and storage time (D), as well as several interaction effects among these factors.

**Table 2:** Trace elements (μg/g dw) of ‘Sauvignon blanc’ and ‘Malbec’ pomace (SBP and MAP, respectively) after gamma irradiation and storage.

Pomace matrix	Irradiation dose (Gy)	Fe		Mn		Zn		Cu		B	
		Day 1	Day 30	Day 1	Day 30	Day 1	Day 30	Day 1	Day 30	Day 1	Day 30
SBP	0	78.0 ± 1.5	81.6 ± 5.1	27.4 ± 0.3	29.3 ± 2.9	3.5 ± 0.7	4.0 ± 0.8	4.0 ± 1.4	4.7 ± 1.3	18.4 ± 1.2	22.0 ± 0.7
SBP	500	85.9 ± 3.9	93.7 ± 4.3	36.4 ± 6.2	33.2 ± 8.4	3.8 ± 1.6	3.8 ± 1.6	3.5 ± 0.8	3.9 ± 1.4	26.1 ± 1.4	21.2 ± 0.8
SBP	1000	77.7 ± 8.3	81.2 ± 12.5	30.9 ± 1.4	24.5 ± 2.2	4.0 ± 1.5	3.8 ± 1.5	4.5 ± 1.2	5.3 ± 2.8	23.3 ± 1.2	24.8 ± 1.6
SBP	1500	97.8 ± 14.8	103.5 ± 11.6	30.5 ± 0.7	32.2 ± 1.5	3.3 ± 0.4	2.8 ± 0.3	3.2 ± 0.2	3.9 ± 0.7	22.4 ± 2.3	24.4 ± 1.8
SBP	2000	67.4 ± 8.1	69.3 ± 4.0	30.0 ± 6.5	34.1 ± 1.4	3.8 ± 1.5	5.1 ± 2.0	3.3 ± 1.5	3.5 ± 0.7	24.0 ± 2.5	25.5 ± 1.7
MAP	0	147.9 ± 10.6	164.4 ± 17.1	16.4 ± 3.4	11.4 ± 2.2	5.3 ± 1.5	6.8 ± 1.2	6.2 ± 0.5	7.2 ± 1.4	25.5 ± 2.6	28.1 ± 1.72
MAP	500	140.4 ± 16.0	120.6 ± 12.4	14.1 ± 2.2	13.3 ± 1.5	5.3 ± 1.5	6.7 ± 1.6	8.3 ± 0.9	6.9 ± 1.4	24.8 ± 1.6	25.8 ± 2.58
MAP	1000	121.1 ± 1.4	142.8 ± 8.8	14.6 ± 0.9	16.5 ± 1.7	6.8 ± 1.5	7.8 ± 2.4	8.0 ± 2.1	7.2 ± 1.3	25.4 ± 3.2	26.3 ± 5.12
MAP	1500	128.5 ± 14.4	132.1 ± 4.3	12.5 ± 0.7	14.0 ± 1.4	4.8 ± 1.4	6.1 ± 0.8	7.3 ± 1.3	7.8 ± 1.6	22.1 ± 1.44	25.2 ± 1.47
MAP	2000	131.4 ± 12.5	132.8 ± 9.3	13.6 ± 1.2	14.5 ± 1.9	5.3 ± 1.5	5.7 ± 1.9	7.0 ± 0.7	8.6 ± 1.5	23.3 ± 3.77	23.1 ± 3.67
Model		***		***		***		***		**	
Irradiation dose (I)		***		n.s.		n.s.		n.s.		n.s.	
Pomace matrix (M)		***		***		***		***		**	
Storage time (D)		*		n.s.		*		n.s.		*	
I × M		***		**		n.s.		n.s.		***	
I × D		n.s.		n.s.		n.s.		n.s.		*	
M × D		n.s.		n.s.		n.s.		n.s.		n.s.	
I × M × D		*		*		n.s.		n.s.		n.s.	

Values are expressed as mean ± SD (*n* = 4). SBP, Sauvignon blanc pomace; MAP, Malbec pomace; dw, dry weight. Data were subjected to three-way ANOVA within a randomised complete block design, with irradiation dose (I), pomace matrix (M), and storage time after irradiation (D) as fixed factors. Significance symbols refer to raw *p*-values from the three-way ANOVA: n.s., not significant; *, *p* < 0.05; **, *p* < 0.01; ***, *p* < 0.001.

Irradiation significantly affected the concentrations of *Fe*, *Mn*, and *Cu*, whereas pomace type exerted a significant effect on all trace elements analysed. Storage time after irradiation was a relevant factor for *Fe*, *Zn*, and *B*. In addition to these main effects, significant interaction terms were detected, particularly for I × M and I × D, indicating that the response of trace elements to gamma-IR depended on both the pomace matrix and the time elapsed after treatment.

The concentrations of *Fe*, *Zn*, *Cu*, and *B* were significantly higher in MAP than in SBP, except for *Mn*, which showed higher levels in SBP. These differences highlight the strong influence of matrix-related characteristics, including cultivar and processing background, on trace element composition.

At the element-specific level, SBP showed a tendency towards increased *B* and *Mn* contents with increasing irradiation dose, particularly after 30 days of storage, although no strictly linear dose–response pattern was observed. In MAP, *Cu* and *Mn* displayed a gradual increase following gamma-IR, whereas *Fe* content tended to decrease with increasing dose, especially at day 30. Boron (*B*) remained relatively stable across irradiation doses and storage times in MAP, while *Zn* showed a slight increase at low to moderate doses, particularly up to 1000 Gy.

Hence, these results indicate that gamma-IR induces moderate, element-specific changes in the trace mineral composition of grape pomace, with responses strongly dependent on pomace type and post-irradiation storage time. The more pronounced differences between SBP and MAP reinforce the relevance of matrix characteristics, likely linked to varietal and processing-related factors, in modulating the effects of irradiation on trace element stability. In this regard, although to date, changes in the mineral content of grape pomace after gamma-IR treatments have not been completely addressed, a previous study conducted by Gülcü et al. (2019) analysed the mineral content of grape pomace, finding 180.8 μg/g dw of *Fe*, 8.17 μg/g dw of *Mn*, and 9.8 μg/g dw of *Zn*, among other trace elements (Gülcü et al., 2019). These values were slightly different to those obtained in this study, emphasising the variability among pomace materials and the close dependence on the variety under study.

These differences support the interpretation that mineral responses to gamma irradiation should be understood as matrix-dependent changes in recoverable mineral concentration rather than as absolute changes in elemental content.

### 3.3 Phenolic profiling variations of grape pomace after gamma radiation storage time

The identified phenolic compounds in the two grape pomaces are presented in Table 3, Table 4, providing insights into the diversity of non-coloured and coloured (poly)phenols present in the grape pomace of the two matrices under study. These tables present the chromatographic and mass spectral information intended for compound identification, whereas the quantitative concentrations of the main phenolic compounds are reported in Table 5, Table 6.

**Table 3:** Identification of non-coloured phenolic profile in grape pomace extract from ‘Sauvignon blanc’ and ‘Malbec’ (SBP and MAP, respectively) exposed to *gamma*-*irradiation* identified by HPLC-DAD-ESI-MSn operated in the negative ionisation mode.

Phenolic compound	Rt (min)	MS[M-H]	MS2[M-H]	MS3[M-H]	SBP	MAP
*Phenolic acids*						
Galloyl hexoside	8.6	331	169,125	125	X	<LOD
Gallic acid	8.9	169	125	–	<LOD	X
Procyanidin trimer (B-type)	9.5	865	695,577,407,363,287,150	289,179	X	X
Unknown	9.8	391	241	213	<LOD	X
Galloyl hexoside	13.0	331	169,125	125	X	X
Protocatechuic acid hexoside	13.4	315	152,109	109	X	X
Galloyl shikimic acid	14.4	325	169	125	X	<LOD
Unknown phenolic acid (isomer 2)	17.1	616	441,271,484	179,271	X	X
*Trans*-caftaric acid (caffeoyl-tartaric acid)	18.3	623	311	149	X	<LOD

*Proanthocyanidins and catechin derivatives*						
Epigallocatechin-epicatechin	14.8	593	423,441,305,467	297	X	X
Proanthocyanidin dimer (B-type) (isomer 1)	17.3	577	289	245,205	<LOD	X
Proanthocyanidin dimer (B-type) (isomer 2)	17.7	577	289	245,205	<LOD	X
Proanthocyanidin dimer (B-type) (isomer 3)	18.8	577	289	245,205	X	X
Proanthocyanidin dimer (B-type) (isomer 4)	19.4	577	289	245,205	X	X
Proanthocyanidin dimer (B-type) (isomer 5)	19.8	577	289	245,205	X	<LOD
(+)-Catechin	21.1	289	245,205	–	X	X
Procyanidin trimer (B-type) (isomer 2)	21.8	865	695,577,407,363,287,150	289,179	X	<LOD
Procyanidin trimer (B-type) (isomer 3)	22.2	865	695,577,407,363,287,150	289,179	X	X
Procyanidin trimer (B-type) (isomer 4)	22.6	865	695,577,407,363,287,150	289,179	X	X
Procyanidin trimer (B-type) (isomer 5)	23.1	865	695,577,407,363,287,150	289,179	X	<LOD
Procyanidin tetramer (B-type)	23.5	1153	863,739,575,449	–	X	<LOD
Proanthocyanidin dimer (B-type) (isomer 7)	24.8	577	289	245,205	<LOD	X
Proanthocyanidin dimer (B-type) (isomer 8)	25.4	577	289	245,205	X	<LOD
Proanthocyanidin dimer (B-type) (isomer 9)	25.7	577	289	245,205	X	X
Proanthocyanidin trimer monogallate (isomer 1)	26.4	1017	729,577,407,287	–	<LOD	X
Proanthocyanidin trimer monogallate (isomer 2)	26.7	1017	729,577,407,287	–	X	X
(−)-Epicatechin	27.3	289	245,293	202,226	X	X
Proanthocyanidin dimer gallate (isomer 1)	28.5	729	577,407,289	407,289	X	<LOD
Procyanidin trimer (B-type) (isomer 6)	29.3	865	695,577,407,363,287,150	289,179	<LOD	X
Proanthocyanidin dimer gallate (isomer 2)	29.8	729	577,407,289	407,289	<LOD	X
Procyanidin trimer (B-type) (isomer 7)	30.5	865	695,577,407,363,287,150	289,179	X	<LOD
Proanthocyanidin dimer gallate (isomer 3)	31.5	729	577,407,289	407,289	X	<LOD
Epicatechin gallate	35.0	441	289,169	245	X	<LOD

*Stilbenes*						
*Trans*-resveratrol	44.8	227	185, 143	–	<LOD	X

*Flavonols*						
Myricetin 3-*O-*glucoside	32.9	479	317	271	<LOD	X
Quercetin 3-*O*-glucoside	36.0	463	301	179,270,151	X	X
Quercetin 3-*O*-glucuronide	36.7	477	301	179,272,151	X	X
Unknown flavonol derivative	38.9	593	285	257	X	<LOD
Kaempferol 3-*O*-glucoside	39.1	447	284	255	X	<LOD
Kaempferol 3-*O*-glucoside (isomer)	41.0	447	285	254	X	X
Kaempferol 3-*O*-glucuronide	41.5	461	285	256	X	<LOD

SBP is unfermented ‘Sauvignon blanc’ pomace; MAP is fermented ‘Malbec’ pomace. X, detection. <LOD, lower than the limit of detection.

**Table 4:** Identification of anthocyanin profile in grape pomace extract from ‘Malbec’ pomace matrix (MAP) exposed to *gamma*-*irradiation* identified by HPLC-DAD-ESI-MSn operated in the positive ionisation mode.

Anthocyanins	Rt (min)	MS[M + H]	MS2[M + H]	MS3[M + H]
Delphinidin 3-*O*-glucoside	24.1	465	303	257
Cyanidin 3-*O-*glucoside	27.0	449	287	193, 231,270
Petunidin 3-*O*-glucoside	28.2	479	317	301,227
Peonidin 3*-O*-glucoside	30.9	463	301	286
Malvidin 3-*O*-glucoside	31.4	493	331	315,179
Delphinidin 3-*O*-acetylglucoside	32.8	507	303	256,149
Petunidin 3-*O*-acetylglucoside	35.3	521	317	301
Peonidin 3-*O*-acetylglucoside	37.5	505	301	285,258
Malvidin 3-*O*-acetylglucoside	38.7	535	331	315,179
Delphinidin 3-*O*-*p*-coumaroylglucoside	40.1	611	303	257,229,149
Malvidin 3-*O*-(6″-caffeoyl)-glucoside	42.6	655	331	315,287,298
Cyanidin 3-*O*-*p*-coumaroylglucoside	43.7	595	287	230,258,189
Petunidin 3-*O*-*p*-coumaroylglucoside	44.4	625	317	301,273
Peonidin 3-*O*-*p*-coumaroylglucoside	48.6	609	301	285
Malvidin 3-*O*-*p*-coumaroylglucoside	49.4	639	331	298,315

MAP, fermented Malbec pomace.

**Table 5:** Content of non-coloured phenolic compounds (μg/g dw) in ‘Sauvignon blanc’ and ‘Malbec’ pomace (SBP and MAP, respectively) after gamma irradiation and storage.

Phenolic compound	Day	SBP						MAP				
		0	500	1000	1500	2000		0	500	1000	1500	2000
*Hydroxybenzoic acid*												
Gallic acid	1	5.99 ± 0.37	5.36 ± 0.77	5.91 ± 1.01	6.71 ± 1.48	5.73 ± 0.96		14.32 ± 0.63	13.95 ± 0.78	13.88 ± 1.04	13.69 ± 0.86	13.80 ± 1.57
	30	5.46 ± 1.38	6.15 ± 0.41	6.34 ± 0.61	6.08 ± 1.37	3.25 ± 0.51		12.05 ± 1.14	11.42 ± 1.27	11.64 ± 1.16	11.49 ± 1.74	10.85 ± 0.82
Syringic acid	1	<LOQ	<LOQ	<LOQ	<LOQ	<LOQ		43.57 ± 4.71	39.76 ± 3.61	42.70 ± 5.04	45.88 ± 9.57	39.98 ± 4.35
	30	<LOQ	<LOQ	<LOQ	<LOQ	<LOQ		44.05 ± 9.62	35.69 ± 7.17	35.43 ± 4.36	36.20 ± 5.31	33.95 ± 2.30
Caffeic acid	1	<LOQ	<LOQ	<LOQ	<LOQ	<LOQ		1.08 ± 0.24	0.67 ± 0.11	<LOQ	<LOQ	<LOQ
	30	<LOQ	<LOQ	<LOQ	<LOQ	<LOQ		0.97 ± 0.05	0.74 ± 0.24	0.90 ± 0.11	0.64 ± 0.32	0.88 ± 0.40
Caftaric acid	1	149.45 ± 19.96	153.21 ± 10.01	141.31 ± 10.32	150.32 ± 51.95	142.64 ± 13.79		<LOQ	<LOQ	<LOQ	<LOQ	<LOQ
	30	139.92 ± 9.00	142.64 ± 15.65	136.13 ± 27.03	139.43 ± 26.98	125.47 ± 12.09		<LOQ	<LOQ	<LOQ	<LOQ	<LOQ
*p*-Coumaric acid	1	<LOQ	<LOQ	<LOQ	<LOQ	<LOQ		17.29 ± 3.41	12.70 ± 1.93	13.87 ± 2.09	13.04 ± 0.82	13.37 ± 2.21
	30	<LOQ	<LOQ	<LOQ	<LOQ	<LOQ		13.89 ± 2.79	12.36 ± 2.15	11.66 ± 1.05	13.11 ± 3.62	9.81 ± 2.50
Ferulic acid	1	<LOQ	<LOQ	<LOQ	<LOQ	<LOQ		9.52 ± 0.50	7.62 ± 1.25	5.69 ± 1.31	4.44 ± 0.85	4.39 ± 0.91
	30	<LOQ	<LOQ	<LOQ	<LOQ	<LOQ		4.69 ± 1.35	4.41 ± 1.24	1.17 ± 0.23	1.17 ± 0.19	1.17 ± 0.45
Fertaric acid	1	8.67 ± 1.59	8.02 ± 0.56	8.43 ± 1.40	11.53 ± 4.77	15.48 ± 5.49		<LOQ	<LOQ	<LOQ	<LOQ	<LOQ
	30	11.83 ± 2.70	8.91 ± 1.73	9.98 ± 2.31	12.63 ± 5.03	6.81 ± 0.52		<LOQ	<LOQ	<LOQ	<LOQ	<LOQ

*Proanthocyanidins and catechin derivatives*												
Catechin	1	144.88 ± 17.46	153.05 ± 10.95	137.47 ± 4.52	141.53 ± 48.67	132.16 ± 23.79		88.56 ± 6.94	79.01 ± 6.56	82.19 ± 10.65	92.82 ± 15.63	84.79 ± 10.60
	30	135.33 ± 7.83	130.21 ± 13.09	115.60 ± 14.70	124.76 ± 19.54	111.70 ± 9.40		74.66 ± 15.35	65.77 ± 11.99	65.88 ± 11.39	71.55 ± 9.34	65.97 ± 8.54
Epicatechin	1	129.45 ± 21.21	124.12 ± 2.08	111.29 ± 12.18	108.37 ± 16.30	107.77 ± 17.55		44.77 ± 2.73	41.76 ± 2.91	43.06 ± 5.47	47.03 ± 5.64	43.41 ± 3.47
	30	108.66 ± 4.90	99.76 ± 8.45	96.62 ± 18.41	95.41 ± 20.55	97.87 ± 9.21		36.50 ± 6.24	35.89 ± 3.04	31.50 ± 5.46	34.58 ± 5.30	29.26 ± 4.67
Epicatechin gallate	1	22.68 ± 4.69	22.33 ± 1.61	20.80 ± 3.23	20.40 ± 6.19	18.47 ± 1.93		10.82 ± 3.54	7.36 ± 0.24	7.49 ± 0.53	7.61 ± 1.42	6.87 ± 0.81
	30	18.42 ± 0.82	18.90 ± 1.34	18.41 ± 3.60	18.13 ± 3.80	16.06 ± 1.99		7.91 ± 1.50	7.90 ± 0.74	6.66 ± 1.01	6.27 ± 1.06	6.11 ± 0.25

*Flavonols*												
Kaempferol 3-*O*-glucoside	1	326.15 ± 53.36	252.94 ± 46.69	258.67 ± 25.48	306.37 ± 82.29	293.56 ± 36.35		<LOQ	<LOQ	<LOQ	<LOQ	<LOQ
	30	315.23 ± 32.37	223.71 ± 59.55	335.98 ± 125.68	303.56 ± 77.91	233.90 ± 6.36		<LOQ	<LOQ	<LOQ	<LOQ	<LOQ
Quercetin 3-*O*-glucoside	1	704.57 ± 43.16	774.22 ± 46.96	745.64 ± 95.75	883.92 ± 159.13	847.87 ± 136.35		155.78 ± 17.71	144.95 ± 21.83	140.16 ± 19.91	145.62 ± 9.52	146.45 ± 11.94
	30	890.38 ± 83.18	821.52 ± 96.13	848.12 ± 239.68	843.35 ± 218.05	544.66 ± 84.97		146.62 ± 22.47	136.24 ± 18.84	117.33 ± 17.67	141.72 ± 41.70	119.84 ± 26.37
Quercetin 3-*O*-glucuronide	1	1056.85 ± 64.74	1161.32 ± 70.44	1118.46 ± 143.63	1325.88 ± 238.69	1271.81 ± 204.53		158.51 ± 10.40	147.59 ± 15.32	155.10 ± 10.94	148.20 ± 6.55	147.20 ± 15.37
	30	1273.08 ± 155.84	1132.28 ± 113.99	1372.18 ± 336.63	1365.03 ± 235.70	816.99 ± 127.46		166.89 ± 19.42	147.95 ± 25.78	133.74 ± 14.66	133.16 ± 34.40	118.46 ± 22.05
Quercetin derivative	1	56.07 ± 21.57	55.05 ± 12.51	46.30 ± 9.06	63.97 ± 27.59	54.42 ± 8.90		<LOQ	<LOQ	<LOQ	<LOQ	<LOQ
	30	68.01 ± 10.31	52.25 ± 7.54	63.41 ± 20.87	63.29 ± 25.66	37.48 ± 5.28		<LOQ	<LOQ	<LOQ	<LOQ	<LOQ

*Stilbenes*												
*Trans*-Resveratrol	1	<LOQ	<LOQ	<LOQ	<LOQ	<LOQ		2.87 ± 0.55	<LOQ	<LOQ	1.60 ± 0.90	1.83 ± 1.55
	30	<LOQ	<LOQ	<LOQ	<LOQ	<LOQ		1.89 ± 0.84	1.84 ± 0.66	<LOQ	1.53 ± 0.89	2.41 ± 0.96

*Total non-coloured phenolics*												
	1	2604.76 ± 91.27	2709.61 ± 179.55	2594.27 ± 264.53	3019.02 ± 380.70	2889.91 ± 372.09		547.10 ± 36.94	495.37 ± 49.76	504.12 ± 46.67	519.22 ± 39.51	502.08 ± 44.21
	30	2966.31 ± 256.32	2636.33 ± 164.99	3002.77 ± 609.20	2971.67 ± 501.41	1994.20 ± 192.16		510.11 ± 72.64	460.22 ± 61.91	415.91 ± 46.14	451.41 ± 96.41	398.70 ± 55.51

SBP, unfermented ‘Sauvignon blanc’ pomace; MAP, fermented ‘Malbec’ pomace.Values are expressed as mean ± SD (*n* = 4). Day: days after irradiation. <LOD, lower than the limit of detection. <LOQ, lower than the limit of quantification. For each pomace matrix, the effects of irradiation dose, storage time, and their interaction were evaluated using a two-way ANOVA within a randomised complete block design. The corresponding ANOVA results are presented in Tables S3 and S4.

**Table 6:** Content of coloured phenolic compounds (anthocyanins) (μg/g dw) in ‘Malbec’ pomace (MAP) after gamma irradiation and storage.

Phenolic compound	Day	0	500	1000	1500	2000
Delphinidin 3-*O*-glucoside	1	415.86 ± 12.56	385.41 ± 41.18	431.67 ± 71.05	384.12 ± 23.76	380.08 ± 66.48
	30	317.92 ± 61.52	335.69 ± 85.62	374.34 ± 65.86	380.56 ± 72.88	332.12 ± 46.28
Cyanidin 3-*O*-glucoside	1	13.61 ± 3.04	11.38 ± 1.57	19.75 ± 3.36	12.36 ± 1.19	12.49 ± 1.52
	30	15.93 ± 1.81	13.48 ± 0.97	13.24 ± 1.61	11.33 ± 1.40	11.12 ± 0.17
Petunidin 3-*O*-glucoside	1	295.11 ± 16.31	273.02 ± 31.02	306.19 ± 59.97	262.91 ± 21.17	274.83 ± 35.79
	30	248.72 ± 41.43	273.81 ± 30.39	278.59 ± 24.93	251.12 ± 70.88	227.63 ± 26.46
Malvidin 3-*O*-glucoside	1	2004.13 ± 108.71	1862.35 ± 213.02	2051.62 ± 363.50	1817.41 ± 142.96	1857.77 ± 294.81
	30	1440.12 ± 286.65	1794.67 ± 179.13	1735.62 ± 128.86	1671.18 ± 455.21	1558.88 ± 107.80
Delphinidin 3-*O*-acetylglucoside	1	88.87 ± 3.52	82.77 ± 7.88	86.70 ± 9.53	82.99 ± 3.76	87.66 ± 11.18
	30	64.01 ± 13.45	81.46 ± 4.22	81.10 ± 5.85	74.85 ± 16.49	68.75 ± 12.30
Petunidin 3-*O-*acetylglucoside	1	83.90 ± 2.12	80.08 ± 10.97	76.94 ± 4.84	61.45 ± 3.18	64.47 ± 10.33
	30	58.35 ± 3.05	66.58 ± 2.56	65.36 ± 5.03	57.37 ± 10.11	56.01 ± 5.44
Peonidin 3-*O*-acetylglucoside	1	48.01 ± 1.52	43.30 ± 6.30	48.83 ± 7.98	38.53 ± 2.05	40.14 ± 7.05
	30	28.84 ± 6.82	40.15 ± 7.21	35.40 ± 7.88	36.04 ± 10.40	32.43 ± 2.27
Malvidin 3-*O*-acetylglucoside	1	636.99 ± 31.42	581.18 ± 70.22	651.44 ± 114.95	571.35 ± 32.42	577.03 ± 87.23
	30	535.06 ± 57.40	590.11 ± 36.38	536.92 ± 46.10	536.84 ± 121.73	459.68 ± 59.43
Cyanidin 3-*O*-p-coumaroylglucoside	1	53.09 ± 4.96	49.71 ± 6.13	50.35 ± 6.68	48.16 ± 1.53	45.85 ± 4.07
	30	32.83 ± 10.54	44.47 ± 4.53	44.92 ± 8.69	40.86 ± 9.52	34.06 ± 7.20
Petunidin 3-*O*-*p* coumaroylglucoside	1	63.37 ± 6.99	57.50 ± 8.47	60.46 ± 13.05	51.09 ± 4.31	51.69 ± 10.64
	30	64.57 ± 13.60	76.34 ± 15.75	54.27 ± 12.96	59.84 ± 15.50	46.19 ± 4.82
Peonidin 3-*O*-*p* coumaroylglucoside	1	14.29 ± 2.37	13.76 ± 3.66	15.74 ± 4.44	13.49 ± 2.90	14.66 ± 5.00
	30	14.37 ± 2.65	18.01 ± 0.35	14.48 ± 1.13	17.20 ± 4.33	13.37 ± 0.71
Malvidin 3-*O*-*p* coumaroylglucoside	1	640.35 ± 56.20	601.72 ± 74.07	648.73 ± 110.41	555.99 ± 38.86	574.60 ± 96.52
	30	665.44 ± 116.66	799.74 ± 123.02	582.21 ± 137.41	584.17 ± 151.86	455.18 ± 64.17
Total Coloured Phenolics	1	4357.58 ± 233.09	4042.18 ± 458.20	4448.44 ± 741.93	3899.85 ± 259.56	3981.30 ± 617.71
	30	3486.17 ± 542.53	4134.51 ± 424.33	3816.46 ± 417.99	3721.37 ± 916.21	3295.42 ± 324.86

These measurements correspond to four replications of each grape pomace (dw). Day: days after irradiation. The effects of irradiation dose, storage time, and their interaction were evaluated using a two-way ANOVA within a randomised complete block design, with source bag included as the blocking factor. The corresponding ANOVA results are presented in Table S4.

Thus, the profiling of non-coloured phenolic compounds by HPLC-DAD-ESI-MSⁿ (Table 3) revealed a complex phenolic composition in grape pomace extracts, mainly characterised by the presence of phenolic acids, proanthocyanidins and catechin derivatives, flavonols, and stilbenes. Phenolic acids included galloyl derivatives, protocatechuic acid conjugates, and caffeoyl-tartaric acid derivatives, which were detected in both pomace types, although with a broader distribution in Sauvignon blanc pomace (SBP).

Proanthocyanidins and catechin derivatives represented the most abundant and diverse class, comprising multiple B-type procyanidin dimers, trimers, and higher oligomers, together with (+)-catechin, (−)-epicatechin, and epicatechin gallate. These compounds were widely detected in SBP, whereas MAP showed a comparatively lower occurrence of higher-degree oligomers (Table 3).

Flavonols, mainly quercetin and kaempferol glycosides and glucuronides, were more frequently detected in MAP than in SBP, highlighting varietal differences in flavonol composition. In addition, the stilbene trans-resveratrol was exclusively detected in MAP samples. Therefore, the phenolic profile observed is consistent with previous reports describing grape pomace as a rich source of oligomeric flavan-3-ols, phenolic acids, and flavonols, with qualitative differences strongly influenced by grape variety and processing conditions (Table 3).

In addition to non-coloured phenolics, anthocyanins were identified in MAP, consistent with the fermented red grape pomace matrix. These compounds were not detected in SBP, as expected for the unfermented ‘Sauvignon blanc’ pomace matrix (Table 4). The anthocyanin profile agreed with previous descriptions in the literature, exhibiting the presence of glucoside, acetylglucoside, and coumaroyl glucoside derivatives (Costa-Pérez et al., 2023; Fontana et al., 2013).

The quantitative analysis of the main phenolic compounds in grape pomace (Table 5, Table 6) revealed marked differences between pomace types, irradiation doses, and storage time. In MAP, malvidin 3-*O*-glucoside was the most abundant anthocyanin, reaching 2004.13 μg/g in control samples at day 1. However, a general decrease in anthocyanin concentration was observed after 30 days of storage, particularly in the control and high-dose treatments, indicating a limited stability of coloured phenolics over time.

In contrast, SBP showed comparatively higher concentrations and improved stability of several non-coloured phenolic compounds during storage. Notably, quercetin 3-*O*-glucuronide reached its maximum concentration at day 30 under 1000 Gy (1372.18 μg/g), while remaining significantly lower in MAP. In addition, quercetin glucoside, kaempferol 3-*O*-glucoside, (+)-catechin and (−)-epicatechin were consistently detected in SBP at both sampling times, showing only moderate variations after 30 days, particularly at intermediate irradiation doses (Table 5).

Both (+)-catechin and (−)-epicatechin were present in both pomace types, whereas syringic acid was the predominant phenolic acid in MAP, reaching values of up to 45.88 μg/g at day 1 under 1500 Gy, and remaining at comparable levels after 30 days. *Trans*-Resveratrol was detected exclusively in MAP, although at relatively low concentrations, and showed limited changes during storage. In addition, in MAP, ferulic acid showed one of the most pronounced decreases, particularly after storage at irradiated treatments. This behaviour may reflect the high sensitivity of hydroxycinnamic acids to oxidative reactions and their possible involvement in polymerisation or binding reactions within the pomace matrix during storage (Table 5).

The total content of non-coloured phenolics further supported the greater stability of SBP, which exhibited its highest values at day 30 under 1000 Gy, whereas MAP showed lower total phenolic contents after storage, particularly in the control and extreme irradiation treatments (0 and 2000 Gy). Similarly, the total anthocyanin content in MAP was better preserved at intermediate irradiation doses, while greater losses were associated with prolonged storage (Table 6).

The effect of gamma-IR on grape pomace phenolics should not be interpreted as a uniform degradation process. The decreases observed for some compounds, particularly at higher doses or after storage, may be associated with oxidation, polymerisation, or degradation of sensitive phenolics. Conversely, the apparent stability or increase of other compounds may reflect irradiation-induced changes in matrix structure, including partial disruption of cell walls or weakened phenolic–matrix interactions, which could enhance compound release. Therefore, the phenolic response to gamma-IR was dose-, matrix-, storage-, and compound-dependent.

These results indicate that phenolic stability after 30 days of storage is strongly dependent on pomace type and irradiation dose. In comparison, MAP showed a greater susceptibility to phenolic degradation over time, particularly affecting anthocyanins, whereas SBP maintained a comparatively more stable phenolic profile, especially at moderate gamma-IR doses.

The matrix-specific effects of irradiation dose, storage time, and their interaction on individual and total phenolic compounds are summarised in Tables S3 and S4. The post-irradiation days also influenced the content of certain compounds, while irradiation only had a lesser compound-dependent impact. This suggests that grape-type differences and temporal evolution might be more relevant than radiation treatments for most of the (poly)phenols assessed.

After FDR adjustment, storage time significantly affected catechin, epicatechin, and epicatechin gallate in SBP, while significant time × irradiation interactions were detected for fertaric acid, quercetin 3-*O*-glucoside, and quercetin 3-*O*-glucuronide. In MAP, storage time significantly affected several anthocyanins and non-coloured phenolic compounds, whereas irradiation effects were observed for a smaller number of compounds. A significant time × irradiation interaction was detected for cyanidin 3-*O*-glucoside.

The exposure of pomace to gamma-IR can lead to the degradation of specific phenolic compounds, which are sensitive to high-energy radiation (Ageyeva et al., 2021), although in this study irradiation was not the most important factor at the doses used, suggesting comparatively limited compositional effects at the doses evaluated. Other studies suggest that the total phenolic content in grape pomace can change based on radiation doses, with moderate doses potentially enhancing phenolic extraction while higher doses causing degradation (Augustine et al., 2013). This is important, since in this study we observed that low-to-intermediate doses were associated with comparatively smaller changes in several quantified phenolics, whereas excessive doses could lead to significant reductions in these compounds (Augustine et al., 2013). This is observed at 2000 Gy (sum of compounds) for MAP, which was more sensitive to irradiation.

Another essential aspect to consider is the time post-irradiation. The composition of phenolic compounds can vary over time, with some compounds becoming more stable and others degrading under light and heat conditions following radiation exposure. Research indicates that specific compounds like quercetin and certain tannins might show enhanced stability at optimal radiation levels, whereas red varieties may exhibit greater losses over time, particularly of anthocyanins (Ageyeva et al., 2021).

In addition, other studies have shown that the type of processing conducted before and after irradiation could also impact the phenolic content. For instance, when fermentation is applied after irradiation, it can enhance the extraction of certain phenolic compounds, potentially leading to increased bioactivity (Leni et al., 2023; Vinha et al., 2023). Furthermore, pre-treatment methods, such as enzyme treatments, are known to modulate the accessibility to phenolic compounds, leading to changes in their release and bioavailability after gamma-IR.

The exposure of pomace to gamma-IR can lead to changes in specific phenolic compounds, but these changes are not necessarily expressed as a uniform decrease across all compound classes. Previous studies in grape pomace and other fruit by-product matrices have reported dose-dependent responses, where moderate irradiation doses may favour phenolic extractability or retention, whereas higher doses and storage can promote degradation or transformation of more sensitive compounds (Augustine et al., 2013; Ito et al., 2016; Ognyanov et al., 2022). This dual behaviour may explain why some phenolic compounds remained stable or increased slightly at low-to-intermediate doses, while more sensitive compounds, particularly some anthocyanins and hydroxycinnamic acids, decreased during storage or at higher irradiation doses.

In this study it was observed that white pomace maintained its phenolic composition relatively stable with or without radiation, while red varieties showed notable losses of anthocyanins. However intermediate radiations resulted in more moderate losses of phenolics.

Finally, antioxidant capacity (*e.g.*, DPPH and ORAC) are summarised in Table 7. A significant three-way interaction (*e.g.*, irradiation, pomace type, and storage time) was found for DPPH. For ORAC, significant main effects of irradiation dose, pomace matrix, and storage time were detected, together with a significant pomace matrix × storage time interaction, whereas the irradiation dose × pomace matrix, irradiation dose × storage time, and three-way interactions were not significant.

**Table 7:** Antioxidant activity (μg TE/g dw) of ‘Sauvignon blanc’ and ‘Malbec’ pomace (SBP and MAP, respectively) after gamma irradiation and storage.

Pomace matrix	Irradiation dose (Gy)	DPPH		ORAC	
		Day 1	Day 30	Day 1	Day 30
SBP	0	417,462.0 ± 16,606.4	215,299.0 ± 15,089.1	267,110.0 ± 19,225.9	225,132.0 ± 10,794.8
	500	428,959.0 ± 23,664.0	262,272.0 ± 23,023.7	293,550.0 ± 40,887.7	248,895.0 ± 12,030.8
	1000	430,304.0 ± 8483.7	254,153.0 ± 26,111.7	250,723.0 ± 12,229.4	216,341.0 ± 19,435.4
	1500	415,054.0 ± 24,275.7	200,625.0 ± 30,709.1	255,560.0 ± 14,675.8	219,657.0 ± 21,377.5
	2000	441,588.0 ± 22,640.3	252,510.0 ± 23,888.2	282,320.0 ± 24,658.8	222,461.0 ± 18,973.9
MAP	0	144,614.0 ± 4526.7	132,478.0 ± 5657.5	172,881.0 ± 14,240.0	168,964.0 ± 18,102.2
	500	166,845.0 ± 14,872.7	113,786.0 ± 6580.9	170,668.0 ± 16,368.0	170,250.0 ± 5909.5
	1000	172,145.0 ± 18,463.6	88,558.0 ± 4974.4	161,680.0 ± 15,150.8	134,354.0 ± 11,980.5
	1500	186,800.0 ± 9140.0	129,349.0 ± 2708.6	156,659.0 ± 8394.1	148,362.0 ± 13,285.0
	2000	203,915.0 ± 13,128.8	115,665.0 ± 4606.9	160,588.0 ± 17,074.7	122,254.0 ± 6535.3
*Three-way ANOVA*					
Model		***		***	
I		***		***	
M		***		***	
D		***		***	
I × M		***		n.s.	
I × D		***		n.s.	
M × D		***		**	
I × M × D		***		n.s.	

Values are expressed as mean ± SD of four independent experimental units per treatment combination (n = 4). DPPH, 2,2-diphenyl-1-picrylhydrazyl radical scavenging assay; ORAC, oxygen radical absorbance capacity; TE, Trolox equivalents; dw, dry weight; SBP, unfermented Sauvignon blanc pomace; MAP, fermented Malbec pomace. Data were analysed by three-way ANOVA within a randomised complete block design, considering irradiation dose (I), pomace matrix (M), and storage time (D) as fixed factors. Significance symbols refer to raw *p*-values from the three-way ANOVA: n.s., not significant; *, *p* < 0.05; **, *p* < 0.01; ***, *p* < 0.001.

These results suggest that gamma-IR may modulate the antioxidant activity of pomace, depending on the dose, pomace type, and storage time. Previous studies have evaluated gamma irradiation as a preservation treatment for grape pomace. Augustine et al. (2013) evaluated combined preservation treatments including gamma irradiation (2–6 kGy) and reported effects on microbial quality and anthocyanin retention during low-temperature storage (Augustine et al., 2013). Compared to the previous study, it is important to note that, in our work, the radiation doses applied were lower (from 0.5 to 2.0 kGy).

On the other hand, other authors focused on different grape varieties. They characterised the antioxidant potential of (poly)phenols using white grape by-products (González-Centeno et al., 2013). Previous studies have also reported antioxidant activity in grape pomace and related grape-derived matrices (González-Centeno et al., 2013; Yıldırım et al., 2005) or oxidative stress mitigation (Theagarajan et al., 2019).

The decrease in antioxidant capacity during storage, particularly for DPPH, indicates that phenolic concentration alone does not fully explain the antioxidant response of irradiated pomace. This apparent dissociation may be explained by compound-specific responses, because decreases in some phenolics were partly offset by the stability or greater recoverability of others. Moreover, DPPH and ORAC reflect the overall radical-scavenging behaviour of the extract, which depends on the chemical composition and relative antioxidant contribution of individual compounds rather than on their summed concentration alone. The decline observed in the non-irradiated controls further indicates that storage contributed substantially to this response. Other qualitative transformations, such as oxidation or polymerisation, cannot be excluded, but were not directly assessed in this study.

### 3.4 Multivariate data analysis of grape pomace composition

Phenolic compounds and mineral elements were interpreted together because both may influence the compositional stability and antioxidant behaviour of grape pomace. In particular, mineral elements can participate in redox-related reactions or interact with phenolics through complexation or chelation, potentially affecting phenolic preservation after gamma-IR.

**Fig. 1:**
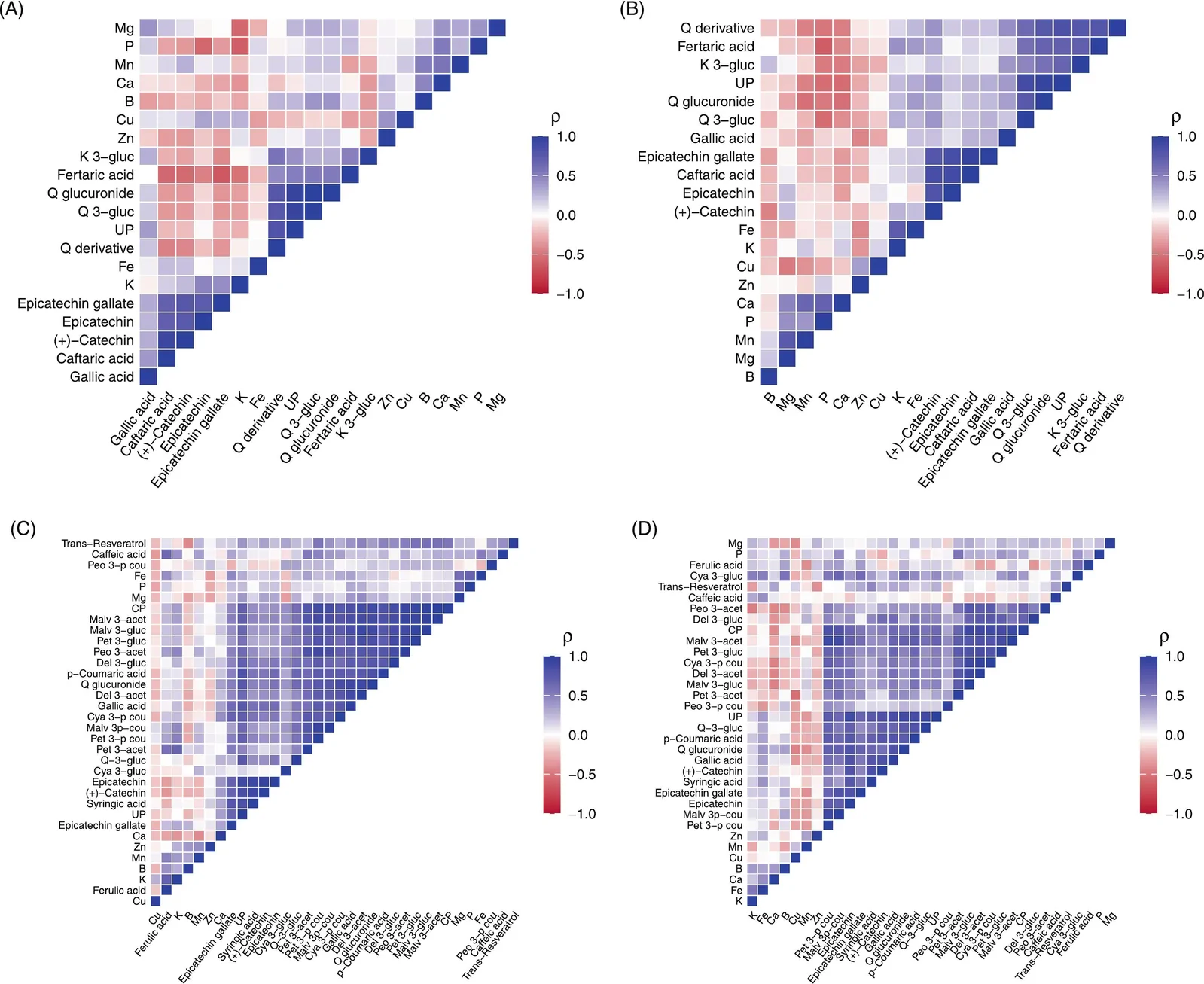
Spearman correlation matrices among analytical variables in unfermented ‘Sauvignon blanc’ pomace (SBP) at day 1 (A) and day 30 (B), and fermented ‘Malbec’ pomace (MAP) at day 1 (C) and day 30 (D) after gamma-IR. Correlations were calculated separately for each pomace matrix and storage time using observations pooled across irradiation doses (*n* = 20; five doses × four experimental blocks). The colour scale represents Spearman's correlation coefficient (ρ), ranging from −1 to +1, with blue indicating positive and red indicating negative correlations. All pairwise correlation coefficients are shown and should be interpreted as exploratory associations. UP, total quantified uncoloured phenolics; CP, total quantified coloured phenolics; Del 3-gluc, delphinidin-3-O-glucoside; Cya 3-gluc, cyanidin-3-O-glucoside; Pet 3-gluc, petunidin-3-O-glucoside; Malv 3-gluc, malvidin-3-O-glucoside; Del 3-acet, delphinidin-3-*O*-acetylglucoside; Pet 3-acet, petunidin-3-*O*-acetylglucoside; Peo 3-acet, peonidin-3-*O*-acetylglucoside; Malv 3-acet, malvidin-3-*O*-acetylglucoside; Cya 3-p-cou, cyanidin-3-O-p-coumaroylglucoside; Pet 3-p-cou, petunidin-3-O-p-coumaroylglucoside; Peo 3-p-cou, peonidin-3-O-p-coumaroylglucoside; Malv 3-p-cou, malvidin-3-O-p-coumaroylglucoside; K 3-gluc, kaempferol-3-O-glucoside; Q 3-gluc, quercetin-3-O-glucoside; Q glucuronide, quercetin 3-O-glucuronide; Q derivative, quercetin derivative; P, phosphorus; K, potassium; Ca, calcium; Mg, magnesium; Fe, iron; Mn, manganese; Zn, zinc; Cu, copper; B, boron. (For interpretation of the references to colour in this figure legend, the reader is referred to the web version of this article.)

Spearman correlation matrices were used to explore associations among phenolic compounds and mineral elements in SBP and MAP at 1 and 30 days after irradiation (Fig. 1). Correlations were calculated separately for each pomace matrix and storage time using observations pooled across the five irradiation doses. Distinct matrix- and time-dependent correlation patterns were observed.

In SBP, positive associations were observed among flavan-3-ols, particularly catechin, epicatechin, and epicatechin gallate, and among flavonol derivatives, including quercetin and kaempferol derivatives. These relationships were maintained, although their relative magnitude varied between days 1 and 30. In MAP, the most apparent pattern was a broad group of positive correlations among anthocyanin derivatives and total anthocyanins at both storage times. Associations between mineral elements and phenolic variables were more variable in direction and magnitude and differed between pomace matrices and storage times. Because all pairwise coefficients are presented, these patterns should be interpreted as exploratory associations rather than evidence of direct chemical or causal relationships.

The correlation matrices showed matrix- and time-dependent patterns of association among phenolic compounds and mineral elements. In SBP, associations were observed between mineral elements (Ca, Mg, Fe, Zn, Mn, Cu, B, P, and K) and phenolic compounds, including gallic acid, flavan-3-ols, and quercetin derivatives. Positive correlations were observed among flavan-3-ols, namely catechin, epicatechin, and epicatechin gallate, as well as between these compounds and phenolic acids such as caftaric acid. In addition, quercetin and kaempferol derivatives (quercetin 3-*O*-glucoside, quercetin 3-*O*-glucuronide, and kaempferol 3-*O*-glucoside) formed a positively correlated cluster. In contrast, the correlation matrices of MAP showed positive associations among several anthocyanins, including malvidin, petunidin, peonidin, and delphinidin derivatives. These patterns indicate coordinated variation among anthocyanin concentrations across irradiation treatments and storage conditions.

Associations between mineral elements and phenolic compounds differed in direction and magnitude according to pomace matrix and storage time. Ca, Mg, and Zn showed positive correlations with some flavan-3-ols and phenolic acids, whereas Cu showed weaker or negative correlations with some phenolic variables. These associations should not be interpreted as evidence that mineral elements directly enhanced phenolic stability or promoted oxidative reactions.

The correlation matrices revealed distinct matrix- and time-dependent patterns of association between phenolic compounds and mineral elements in grape pomace (Fig. 1). These results should be interpreted as exploratory associations rather than evidence of direct chemical or causal interactions.

**Fig. 2:**
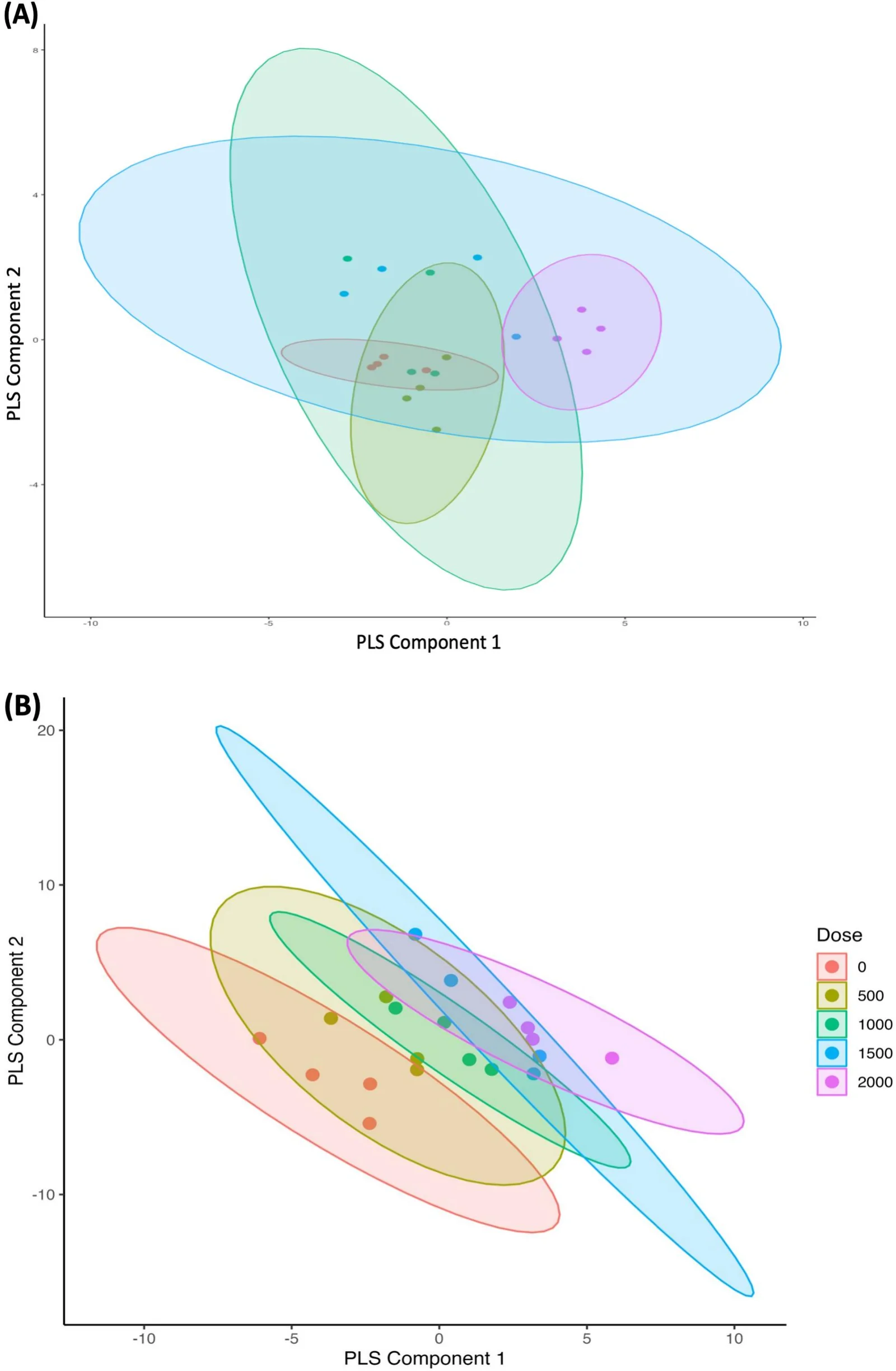
Partial Least Squares Discriminant Analysis (PLS-DA) score plots for grape pomace extracts after gamma-IR and 30 days of storage: (A) unfermented Sauvignon blanc pomace (SBP) and (B) fermented Malbec pomace (MAP). The models included phenolic compounds, mineral elements, and antioxidant-related variables and were fitted using two latent components. Model performance was evaluated using four-fold leave-one-block-out cross-validation. For SBP, R^2^X = 0.425, R^2^Y = 0.419, Q^2^ = 0.221, and classification accuracy = 50%; for MAP, R^2^X = 0.518, R^2^Y = 0.332, Q^2^ = 0.121, and classification accuracy = 40%. Permutation testing using 999 within-block permutations yielded *p* = 0.001 for Q^2^ and *p* = 0.015 for classification accuracy in SBP, and *p* = 0.009 and *p* = 0.067, respectively, in MAP. UP, uncoloured phenolics; CP, coloured phenolics; K 3-O-glc, kaempferol 3-O-glucoside; Q 3-O-glc, quercetin 3-O-glucoside; Q 3-O-glcA, quercetin 3-O-glucuronide; Dp 3-O-glc, delphinidin 3-O-glucoside; Cy 3-O-glc, cyanidin 3-O-glucoside; Pt 3-O-glc, petunidin 3-O-glucoside; Mv 3-O-glc, malvidin 3-O-glucoside; Dp 3-O-acet, delphinidin 3-*O*-acetylglucoside; Pt 3-O-acet, petunidin 3-*O*-acetylglucoside; Pn 3-O-acet, peonidin 3-*O*-acetylglucoside; Mv 3-O-acet, malvidin 3-*O*-acetylglucoside; Cy 3-O-cou, cyanidin 3-O-coumaroylglucoside; Pt 3-O-cou, petunidin 3-O-coumaroylglucoside; Pn 3-O-cou, peonidin 3-O-coumaroylglucoside; Mv 3-O-cou, malvidin 3-O-p-coumaroylglucoside.

Finally, the combined mineral and phenolic datasets were further explored using Partial Least Squares Discriminant Analysis (PLS-DA) to evaluate the overall chemical differentiation among irradiation treatments after 30 days of storage (Fig. 2). The two-component model for SBP explained 42.5% of the variance in the predictor matrix (R^2^X) and 41.9% of the variance in the response matrix (R^2^Y), with a cross-validated Q^2^ of 0.221, a classification accuracy of 50%, and an error rate of 50%. For MAP, the model explained 51.8% of the variance in X and 33.2% of the variance in Y, with a Q^2^ of 0.121, a classification accuracy of 40%, and an error rate of 60%. Block-preserving permutation tests indicated that the observed Q^2^ values were higher than expected by chance for both SBP (*p* = 0.001) and MAP (*p* = 0.009). Nevertheless, the relatively low Q^2^ values and classification accuracies indicate limited predictive capacity; therefore, the PLS-DA score plots were interpreted as exploratory evidence of treatment-related structuring rather than definitive discrimination among irradiation doses.

In “Sauvignon blanc” pomace (Fig. 2A), the exploratory PLS-DA suggested an effect of irradiation on the combined mineral–phenolic profile, with samples showing a progressive separation along Component 1 as irradiation dose increased. This distribution suggested a dose–response pattern, particularly after 30 days, with limited overlap between low and high irradiation treatments. The loading and VIP analyses indicated that flavan-3-ols (catechin, epicatechin, and epicatechin gallate) and phenolic acids (caftaric, gallic, and ferulic acids) contributed to the negative side of Component 1, whereas mineral elements such as *Fe*, *K*, *P*, *Ca*, *Mn*, and *B*, together with selected phenolics including kaempferol derivatives and fertaric acid, emerged as potential discriminating variables. These results suggest a combined contribution of phenolics and minerals as candidate contributors to treatment differentiation after storage.

A comparable irradiation-dependent separation was observed in Malbec pomace (Fig. 2B), the score plot also suggested treatment-related structuring, although the groups showed considerable overlap and the model exhibited limited classification performance. Coloured phenolics, particularly anthocyanins, together with selected mineral elements, contributed prominently to the observed multivariate pattern. These results suggest that the anthocyanin-rich composition of MAP influenced its multivariate response to irradiation and storage, although the exploratory nature of the model should be considered.

For completeness, PLS-DA models corresponding to samples analysed at day 1 are provided in Supplementary Fig. S1. The two-component SBP model showed a Q^2^ of 0.037 and a classification accuracy of 45%, whereas the MAP model showed a Q^2^ of 0.065 and an accuracy of 55%.

**Fig. 3:**
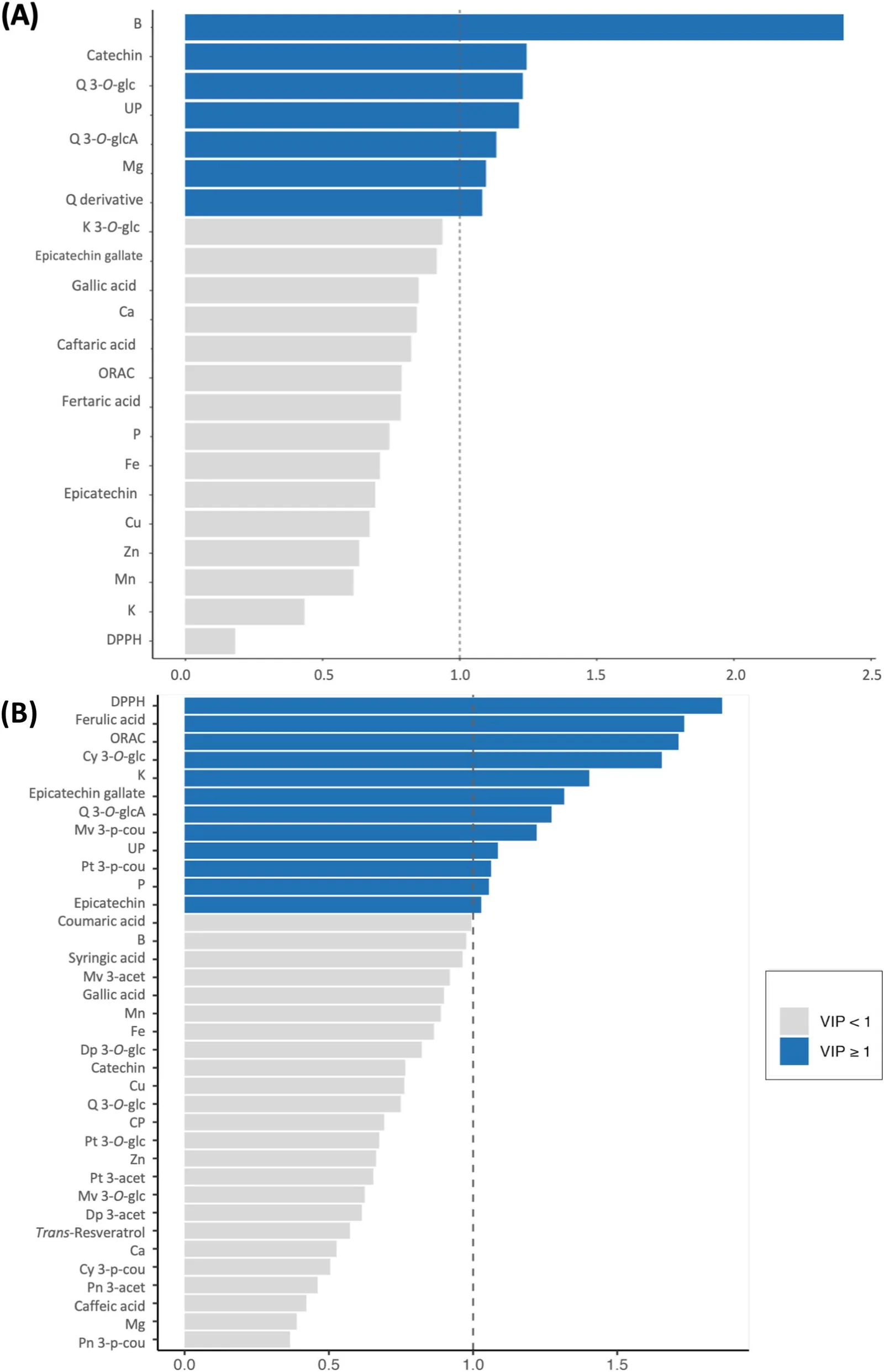
Variable Importance in Projection (VIP) scores derived from the PLS-DA models for grape pomace extracts after gamma-IR and 30 days of storage: (A) unfermented Sauvignon blanc pomace (SBP) and (B) fermented Malbec pomace (MAP). The analysis included phenolic compounds and mineral elements. The dashed line indicates the VIP threshold of 1; variables with VIP scores >1 were considered candidate contributors to the observed treatment-related separation. UP, uncoloured phenolics; CP, coloured phenolics; K 3-O-glc, kaempferol 3-O-glucoside; Q 3-O-glc, quercetin 3-O-glucoside; Q 3-O-glcA, quercetin 3-O-glucuronide; Dp 3-O-glc, delphinidin 3-O-glucoside; Cy 3-O-glc, cyanidin 3-O-glucoside; Pt 3-O-glc, petunidin 3-O-glucoside; Mv 3-O-glc, malvidin 3-O-glucoside; Dp 3-O-acet, delphinidin 3-*O*-acetylglucoside; Pt 3-O-acet, petunidin 3-*O*-acetylglucoside; Pn 3-O-acet, peonidin 3-*O*-acetylglucoside; Mv 3-O-acet, malvidin 3-*O*-acetylglucoside; Cy 3-O-cou, cyanidin 3-O-coumaroylglucoside; Pt 3-O-cou, petunidin 3-O-coumaroylglucoside; Pn 3-O-cou, peonidin 3-O-coumaroylglucoside; Mv 3-O-cou, malvidin 3-O-p-coumaroylglucoside.

To identify candidate variables contributing to the patterns observed in the exploratory PLS-DA models, Variable Importance in Projection (VIP) scores were analysed for both grape pomace types after 30 days of storage (Fig. 3). Only variables with VIP values above the commonly accepted threshold (VIP > 1) were considered candidate contributors to group separation.

In SBP (Fig. 3A), the VIP analysis identified flavan-3-ols, including catechin, epicatechin, and epicatechin gallate, together with phenolic acids such as caftaric, gallic, and fertaric acids, as variables with high VIP scores after 30 days of storage. Several mineral elements, namely K, P, Ca, Fe, Mn, and B, also showed high VIP scores and were therefore considered candidate contributors to the observed treatment-related pattern. Overall, these results suggest that both phenolic and mineral variables contributed to the exploratory multivariate pattern in SBP.

In “Malbec” pomace (Fig. 3B), VIP scores were predominantly driven by coloured phenolics, particularly anthocyanins such as malvidin 3-*O*-glucoside, petunidin 3-*O*-glucoside, delphinidin 3-*O*-glucoside, and their acylated derivatives. Selected flavonols and trans-resveratrol were also identified as candidate contributors to the observed multivariate pattern, together with minerals such as Ca, Mg, Zn, and Fe. The prominence of anthocyanins among the highest VIP-ranked variables is consistent with the strong influence of irradiation and storage on the anthocyanin-rich matrix of MAP after 30 days.

The VIP analysis suggests that the variables contributing to the observed patterns after 30 days differed markedly between pomace matrices, with non-coloured phenolics and minerals among the main candidate contributors in SBP and anthocyanins showing greater prominence in MAP.

For completeness, VIP score plots corresponding to samples analysed at day 1 after irradiation are provided in Supplementary Fig. S2.

Comparison of the day-1 and day-30 models suggested that the multivariate structure became more evident during storage, particularly in SBP, for which Q^2^ increased from 0.037 to 0.221. In MAP, Q^2^ increased only modestly from 0.065 to 0.121, while classification accuracy decreased from 55% to 40%. Therefore, the temporal evolution of treatment-related differentiation was not equally consistent across both pomace matrices.

Hence, white and red grape pomaces exhibited distinct multivariate responses to gamma-IR, reflecting differences in phenolic composition and matrix characteristics. In MAP, anthocyanins and phenolic acids were among the main candidate contributor to the observed treatment-related pattern after storage, whereas in SBP, non-coloured phenolics and mineral elements contributed more strongly to treatment separation. These findings support the notion that phenolic stability and chemical evolution during storage are governed not only by irradiation dose but also by pomace type and storage time.

In the present study, the apparent stability or increase of some phenolic compounds may partly reflect changes in extractability, although this mechanism was not directly evaluated. Mineral–phenolic associations varied between matrices and storage times. P, Ca, Mg, Mn, and Zn showed positive correlations with some phenolic variables, whereas Fe and Cu showed more variable or negative associations in some cases. These patterns should be interpreted as exploratory associations and do not demonstrate synergistic, pro-oxidant, or causal interactions.

The evolution of these relationships over the 30-day storage period was evident in both the PLS-DA models and correlation matrices. In MAP, anthocyanins and selected phenolic acids maintained high VIP scores and coherent associations with specific minerals after storage, indicating a persistent contribution of these compounds to sample discrimination despite overall compositional changes. In contrast, SBP exhibited a greater reorganisation of associations among antioxidant-related variables, consistent with its distinct phenolic composition and matrix characteristics (Bakota et al., 2015).

Hence, the results indicate that gamma irradiation induced dose-, storage-, and matrix-dependent changes in the chemical composition of grape pomace. Intermediate doses (500–1000 Gy) were generally associated with comparatively smaller changes in several phenolic variables, although this response was not uniform across compounds or pomace matrices. Differences between the two bulk lots may reflect their distinct cultivar and processing backgrounds. Although the study was limited to two contrasting commercial pomace matrices from a defined harvest and production context, their distinct responses demonstrate different treatment responses within the materials examined. Future studies incorporating additional vintages and winemaking conditions would help establish the broader consistency of these responses. From a regulatory perspective, the doses evaluated (0.5–2.0 kGy) are below those authorised for several food-irradiation applications internationally. The European Union, for example, permits doses of up to 10 kGy for dried aromatic herbs, spices, and vegetable seasonings, whereas the United States and Australia/New Zealand apply food- and purpose-specific authorisations. Nevertheless, these provisions do not imply automatic authorisation of irradiated grape pomace, which would remain subject to product-specific approval and labelling requirements in each jurisdiction.

## 4 Conclusions

Gamma irradiation induced dose-, matrix-, and storage-dependent changes in the phenolic composition and recoverable mineral concentrations of the two specific commercial grape pomace bulk lots evaluated. Gamma-IR did not produce a simple linear degradation of grape pomace phenolics. Instead, the response depended on irradiation dose, pomace matrix, storage time, and compound class, reflecting both degradation of sensitive compounds and possible changes in phenolic extractability. Intermediate doses (500–1000 Gy) showed the most favourable compositional balance, with comparatively greater phenolic preservation and only minor changes in recoverable mineral profile, whereas higher doses promoted greater losses of oxidation-sensitive phenolics. The distinct responses of Sauvignon blanc and Malbec bulk lots indicate that the effects of gamma irradiation were strongly matrix-dependent under the experimental conditions evaluated. In SBP, the initial irradiation-induced reduction in bacterial load was not sustained during 30 days of refrigerated storage, indicating that gamma-IR did not provide sustained microbiological stabilisation under these conditions. Overall, gamma irradiation shows potential as a pretreatment for modulating the compositional stability of grape pomace. However, further studies should confirm these responses across additional vintages and production conditions and determine whether the observed compositional effects translate into sustained microbiological stability and functional performance in real product systems.

## CRediT authorship contribution statement

**María Dolores López-Belchí:** Writing – original draft, Visualization, Resources, Methodology, Investigation, Funding acquisition, Formal analysis, Conceptualization. **Daniel Villegas:** Writing – review & editing, Methodology, Investigation. **Raúl Domínguez-Perles:** Writing – review & editing, Methodology, Investigation, Formal analysis. **Sonia Medina:** Writing – review & editing, Investigation, Formal analysis. **Cristina García-Viguera:** Writing – review & editing, Investigation, Formal analysis. **Bárbara Hormazabal:** Methodology, Formal analysis. **V. Felipe Laurie:** Writing – review & editing, Resources, Methodology, Investigation, Conceptualization.

## Funding

This work has been supported by FOVI 230048 (ANID, Chile) and FONDECYT REGULAR projects 1240947 and 1231484.

## Declaration of competing interest

The authors declare that they have no known competing financial interests or personal relationships that could have appeared to influence the work reported in this paper.

## Data Availability

Data will be made available on request.
