# Supplementary material for: Gamma irradiation as a grape pomace stabilisation treatment: Assessment of phenolic and mineral preservation
Source: Food Chem X. 2026 Sep 8;39:104435. doi: 10.1016/j.fochx.2026.104435 (PMC13580048; doi:10.1016/j.fochx.2026.104435)
Supplement: Supplementary file 1 [file mmc1.docx]

**Gamma Irradiation as a Grape Pomace Stabilization Treatment: Assessment of Phenolic and Mineral Preservation**

María Dolores López-Belchí^a,b^*; Daniel Villegas^c^; Raúl Domínguez-Perles^d^; Sonia Medina^d^; Cristina García-Viguera^d^; Bárbara Hormazabal^e^; V. Felipe Laurie^e,f^*

^a^ Departamento de Producción Vegetal, Facultad de Agronomía, Universidad de Concepción, 4030000 Concepción, Chile; [mlopezb@udec.cl](mailto:mlopezb@udec.cl)

^b^ Laboratorio de Bioactivos e Ingredientes Vegetales (BIOINVE), Centro de Biotecnología, Universidad de Concepción, 4030000 Concepción, Chile

^c^ Laboratorio de Radiobiología Vegetal, Comisión Chilena de Energía Nuclear; Santiago, Chile; [daniel.villegas@cchen.cl](mailto:daniel.villegas@cchen.cl)

^d^ Lab. Fitoquímica y Alimentos saludables (LabFAS). Dept. of Food Science and Technology, CSIC, CEBAS, Campus Universitario de Espinardo 25, 30100 Murcia, Spain; [rdperles@cebas.csic.es](mailto:rdperles@cebas.csic.es), [smescudero@cebas.csic.es](mailto:smescudero@cebas.csic.es), [cgviguera@cebas.csic.es](mailto:cgviguera@cebas.csic.es)

^e^ Departamento de Horticultura, Facultad de Ciencias Agrarias, Universidad de Talca; [flaurie@utalca.cl](mailto:flaurie@utalca.cl), barbara14moya@gmail.com

^f^ Centro de Investigación e Innovación VitiScience -CIA 250013, Santiago, Chile.

*	Correspondence: [mlopezb@udec.cl](mailto:mlopezb@udec.cl); [flaurie@utalca.cl](mailto:flaurie@utalca.cl)

Table S1**.** Microbial counts (log_10_ CFU/g fw) in unfermented ‘Sauvignon blanc’ pomace (SBP) and fermented ‘Malbec’ pomace (MAP) after gamma irradiation and 1 or 30 days of refrigerated storage.

| Irradiation dose (Gy) | SBP bacteria  Day 1 | SBP bacteria  Day 30 | SBP fungi  Day 1 | SBP fungi  Day 30 | MAP bacteria  Day 1 | MAP bacteria  Day 30 | MAP fungi  Day 1 | MAP fungi  Day 30 |
| --- | --- | --- | --- | --- | --- | --- | --- | --- |
| 0 | 7.52 ± 0.17 | 7.31 ± 0.23 | <LOD | <LOD | <LOD | <LOD | <LOD | <LOD |
| 500 | 6.58 ± 0.68 | 7.48 ± 0.26 | <LOD | <LOD | <LOD | <LOD | <LOD | <LOD |
| 1000 | 6.58 ± 0.68 | 7.45 ± 0.64 | <LOD | <LOD | <LOD | <LOD | <LOD | <LOD |
| 1500 | 6.08 ± 0.15 | 7.70 ± 0.40 | <LOD | <LOD | <LOD | <LOD | <LOD | <LOD |
| 2000 | 4.49 ± 0.31 | 7.74 ± 0.28 | <LOD | <LOD | <LOD | <LOD | <LOD | <LOD |

Values are expressed as mean ± standard deviation (SD) of four independent experimental units (n = 4), after log_10_ transformation. The limit of detection (LOD) of the culture method was 1.0 × 10³ CFU/g fw, equivalent to 3.00 log₁₀ CFU/g fw. Values below this limit are reported as <LOD. SBP, unfermented ‘Sauvignon blanc’ pomace; MAP, fermented ‘Malbec’ pomace; CFU, colony-forming units; fw, fresh weight.

| **Standard** | **Compounds quantified as equivalent** | **λ (nm)** | **Calibration range (µg g^˗1^)** | **R²** | **LOD**  **(µg g^˗1^)** | **LOQ**  **(µg g^˗1^)** |
| --- | --- | --- | --- | --- | --- | --- |
| Catechin | Catechin, epicatechin, and epicatechin gallate | 280 | 3.4-6967.2 | 0.9999 | 1.7 | 3.4 |
| Gallic acid | Hydroxybenzoic acids | 280 | 0.6-290.1 | 0.9972 | 0.2 | 0.6 |
| *p*-Coumaric acid | Hydroxycinnamic acids | 320 | 0.7-615.3 | 0.9989 | 0.2 | 0.7 |
| *trans*-Resveratrol | Stilbenes | 320 | 1.5-380.7 | 0.9899 | 0.7 | 1.5 |
| Quercetin-3-O-rutinoside | Flavonols | 360 | 11.9-3052.5 | 0.9961 | 6.0 | 11.9 |
| Malvidin-3-*O*-glucoside | Anthocyanins | 520 | 8.8-4494.0 | 0.9999 | 4.4 | 8.8 |

Table S2. Calibration parameters for the quantification of phenolic compounds by HPLC-DAD-ESI-MS^n^.

Calibration ranges were established according to the linear response of the analytical method. LOD and LOQ were determined from the chromatographic signal-to-noise ratio and expressed in the same final units used for reporting sample concentrations. LOD, limit of detection; LOQ, limit of quantification.

Table S3. Blocked two-way ANOVA for non-coloured phenolic compounds in unfermented ‘Sauvignon blanc’ pomace (SBP).

| **Response variable** | **Effect** | **df** | **Mean square** | **Residual mean square** | **F** | **Raw p** | **FDR q** |
| --- | --- | --- | --- | --- | --- | --- | --- |
| Gallic acid | Time | 1 | 2.393 | 2.264 | 1.057 | 0.3130 | 0.6886 |
| Gallic acid | Irradiation | 4 | 4.261 | 2.264 | 1.882 | 0.1425 | 0.3134 |
| Gallic acid | Time × Irradiation | 4 | 3.240 | 2.264 | 1.431 | 0.2507 | 0.4714 |
| Caftaric acid | Time | 1 | 1138.0 | 565.52 | 2.012 | 0.1675 | 0.4605 |
| Caftaric acid | Irradiation | 4 | 248.93 | 565.52 | 0.440 | 0.7784 | 0.7784 |
| Caftaric acid | Time × Irradiation | 4 | 36.85 | 565.52 | 0.065 | 0.9917 | 0.9917 |
| Fertaric acid | Time | 1 | 1.558 | 10.96 | 0.142 | 0.7091 | 0.9704 |
| Fertaric acid | Irradiation | 4 | 16.84 | 10.96 | 1.536 | 0.2198 | 0.3288 |
| Fertaric acid | Time × Irradiation | 4 | 44.33 | 10.96 | 4.044 | 0.0107 | **0.0393** |
| (+)-Catechin | Time | 1 | 3346.7 | 448.04 | 7.470 | 0.0109 | **0.0401** |
| (+)-Catechin | Irradiation | 4 | 577.66 | 448.04 | 1.289 | 0.2989 | 0.3288 |
| (+)-Catechin | Time × Irradiation | 4 | 58.45 | 448.04 | 0.130 | 0.9699 | 0.9917 |
| (–)-Epicatechin | Time | 1 | 2734.5 | 223.68 | 12.225 | 0.0016 | **0.0181** |
| (–)-Epicatechin | Irradiation | 4 | 436.34 | 223.68 | 1.951 | 0.1308 | 0.3134 |
| (–)-Epicatechin | Time × Irradiation | 4 | 69.80 | 223.68 | 0.312 | 0.8674 | 0.9917 |
| Epicatechin gallate | Time | 1 | 86.97 | 10.92 | 7.962 | 0.0089 | **0.0401** |
| Epicatechin gallate | Irradiation | 4 | 14.82 | 10.92 | 1.357 | 0.2750 | 0.3288 |
| Epicatechin gallate | Time × Irradiation | 4 | 1.499 | 10.92 | 0.137 | 0.9671 | 0.9917 |
| Kaempferol 3-O-glucoside | Time | 1 | 256.29 | 3941.1 | 0.065 | 0.8006 | 0.9704 |
| Kaempferol 3-O-glucoside | Irradiation | 4 | 8911.2 | 3941.1 | 2.261 | 0.0888 | 0.3134 |
| Kaempferol 3-O-glucoside | Time × Irradiation | 4 | 5195.4 | 3941.1 | 1.318 | 0.2884 | 0.4714 |
| Quercetin 3-O-glucoside | Time | 1 | 26.75 | 19042.6 | 0.001 | 0.9704 | 0.9704 |
| Quercetin 3-O-glucoside | Irradiation | 4 | 28744.3 | 19042.6 | 1.509 | 0.2274 | 0.3288 |
| Quercetin 3-O-glucoside | Time × Irradiation | 4 | 70419.5 | 19042.6 | 3.698 | 0.0159 | **0.0436** |
| Quercetin 3-O-glucuronide | Time | 1 | 254.69 | 35175.5 | 0.007 | 0.9328 | 0.9704 |
| Quercetin 3-O-glucuronide | Irradiation | 4 | 1.018e+05 | 35175.5 | 2.895 | 0.0408 | 0.2510 |
| Quercetin 3-O-glucuronide | Time × Irradiation | 4 | 1.601e+05 | 35175.5 | 4.552 | 0.0061 | **0.0337** |
| Quercetin derivative | Time | 1 | 29.84 | 278.06 | 0.107 | 0.7457 | 0.9704 |
| Quercetin derivative | Irradiation | 4 | 405.16 | 278.06 | 1.457 | 0.2427 | 0.3288 |
| Quercetin derivative | Time × Irradiation | 4 | 357.68 | 278.06 | 1.286 | 0.3000 | 0.4714 |
| Total non-coloured phenolics | Time | 1 | 24260.4 | 1.170e+05 | 0.207 | 0.6525 | 0.9704 |
| Total non-coloured phenolics | Irradiation | 4 | 3.279e+05 | 1.170e+05 | 2.803 | 0.0456 | 0.2510 |
| Total non-coloured phenolics | Time × Irradiation | 4 | 5.477e+05 | 1.170e+05 | 4.682 | 0.0053 | **0.0337** |

The model included storage time, irradiation dose, their interaction, and source bag as the blocking factor. Degrees of freedom were 1 for time, 4 for irradiation dose, 4 for the interaction, and 27 for the residual error. Raw p-values were adjusted using the Benjamini–Hochberg false discovery rate procedure separately for each model term across all estimable response variables within the pomace matrix. FDR-adjusted q-values < 0.05 are shown in bold.

Table S4. Blocked two-way ANOVA for coloured and non-coloured phenolic compounds in fermented ‘Malbec’ pomace (MAP).

| **Response variable** | **Effect** | **df** | **Mean square** | **Residual mean square** | **F** | **Raw p** | **FDR q** |
| --- | --- | --- | --- | --- | --- | --- | --- |
| Delphinidin 3-O-glucoside | Time | 1 | 26318.9 | 3357.7 | 7.838 | 0.0093 | **0.0194** |
| Delphinidin 3-O-glucoside | Irradiation | 4 | 2925.1 | 3357.7 | 0.871 | 0.4940 | 0.5880 |
| Delphinidin 3-O-glucoside | Time × Irradiation | 4 | 2252.5 | 3357.7 | 0.671 | 0.6179 | 0.7724 |
| Cyanidin 3-O-glucoside | Time | 1 | 8.083 | 3.277 | 2.467 | 0.1279 | 0.1523 |
| Cyanidin 3-O-glucoside | Irradiation | 4 | 34.69 | 3.277 | 10.586 | <0.0001 | **0.0003** |
| Cyanidin 3-O-glucoside | Time × Irradiation | 4 | 25.56 | 3.277 | 7.799 | 0.0003 | **0.0065** |
| Petunidin 3-O-glucoside | Time | 1 | 6990.7 | 1599.3 | 4.371 | 0.0461 | 0.0607 |
| Petunidin 3-O-glucoside | Irradiation | 4 | 2068.3 | 1599.3 | 1.293 | 0.2975 | 0.4067 |
| Petunidin 3-O-glucoside | Time × Irradiation | 4 | 893.43 | 1599.3 | 0.559 | 0.6946 | 0.7893 |
| Malvidin 3-O-glucoside | Time | 1 | 7.760e+05 | 63661.4 | 12.189 | 0.0017 | **0.0042** |
| Malvidin 3-O-glucoside | Irradiation | 4 | 50043.6 | 63661.4 | 0.786 | 0.5443 | 0.6185 |
| Malvidin 3-O-glucoside | Time × Irradiation | 4 | 72638.6 | 63661.4 | 1.141 | 0.3585 | 0.5611 |
| Delphinidin 3-O-acetylglucoside | Time | 1 | 1383.9 | 99.11 | 13.963 | 0.0009 | **0.0028** |
| Delphinidin 3-O-acetylglucoside | Irradiation | 4 | 73.45 | 99.11 | 0.741 | 0.5723 | 0.6221 |
| Delphinidin 3-O-acetylglucoside | Time × Irradiation | 4 | 191.51 | 99.11 | 1.932 | 0.1339 | 0.5161 |
| Petunidin 3-O-acetylglucoside | Time | 1 | 1596.7 | 46.93 | 34.022 | <0.0001 | **<0.0001** |
| Petunidin 3-O-acetylglucoside | Irradiation | 4 | 355.11 | 46.93 | 7.566 | 0.0003 | **0.0026** |
| Petunidin 3-O-acetylglucoside | Time × Irradiation | 4 | 129.50 | 46.93 | 2.759 | 0.0481 | 0.4010 |
| Peonidin 3-O-acetylglucoside | Time | 1 | 844.80 | 42.27 | 19.985 | 0.0001 | **0.0005** |
| Peonidin 3-O-acetylglucoside | Irradiation | 4 | 55.30 | 42.27 | 1.308 | 0.2921 | 0.4067 |
| Peonidin 3-O-acetylglucoside | Time × Irradiation | 4 | 100.49 | 42.27 | 2.377 | 0.0769 | 0.4805 |
| Malvidin 3-O-acetylglucoside | Time | 1 | 51659.6 | 5418.6 | 9.534 | 0.0046 | **0.0105** |
| Malvidin 3-O-acetylglucoside | Irradiation | 4 | 7958.6 | 5418.6 | 1.469 | 0.2392 | 0.4067 |
| Malvidin 3-O-acetylglucoside | Time × Irradiation | 4 | 6357.0 | 5418.6 | 1.173 | 0.3447 | 0.5611 |
| Cyanidin 3-O-p-coumaroylglucoside | Time | 1 | 1000.5 | 47.71 | 20.970 | <0.0001 | **0.0004** |
| Cyanidin 3-O-p-coumaroylglucoside | Irradiation | 4 | 79.10 | 47.71 | 1.658 | 0.1888 | 0.3933 |
| Cyanidin 3-O-p-coumaroylglucoside | Time × Irradiation | 4 | 79.72 | 47.71 | 1.671 | 0.1858 | 0.5161 |
| Petunidin 3-O-p-coumaroylglucoside | Time | 1 | 117.01 | 114.38 | 1.023 | 0.3208 | 0.3487 |
| Petunidin 3-O-p-coumaroylglucoside | Irradiation | 4 | 405.33 | 114.38 | 3.544 | 0.0190 | 0.0677 |
| Petunidin 3-O-p-coumaroylglucoside | Time × Irradiation | 4 | 221.73 | 114.38 | 1.939 | 0.1328 | 0.5161 |
| Peonidin 3-O-p-coumaroylglucoside | Time | 1 | 12.03 | 10.59 | 1.136 | 0.2959 | 0.3363 |
| Peonidin 3-O-p-coumaroylglucoside | Irradiation | 4 | 4.615 | 10.59 | 0.436 | 0.7816 | 0.7816 |
| Peonidin 3-O-p-coumaroylglucoside | Time × Irradiation | 4 | 14.52 | 10.59 | 1.371 | 0.2701 | 0.5611 |
| Malvidin 3-O-p-coumaroylglucoside | Time | 1 | 1708.2 | 9815.1 | 0.174 | 0.6798 | 0.6798 |
| Malvidin 3-O-p-coumaroylglucoside | Irradiation | 4 | 41475.5 | 9815.1 | 4.226 | 0.0087 | **0.0365** |
| Malvidin 3-O-p-coumaroylglucoside | Time × Irradiation | 4 | 29232.2 | 9815.1 | 2.978 | 0.0369 | 0.4010 |
| Gallic acid | Time | 1 | 59.50 | 1.425 | 41.766 | <0.0001 | **<0.0001** |
| Gallic acid | Irradiation | 4 | 0.7849 | 1.425 | 0.551 | 0.7000 | 0.7291 |
| Gallic acid | Time × Irradiation | 4 | 0.1967 | 1.425 | 0.138 | 0.9667 | 0.9667 |
| Syringic acid | Time | 1 | 282.60 | 38.24 | 7.391 | 0.0113 | **0.0202** |
| Syringic acid | Irradiation | 4 | 60.90 | 38.24 | 1.593 | 0.2049 | 0.3940 |
| Syringic acid | Time × Irradiation | 4 | 29.28 | 38.24 | 0.766 | 0.5569 | 0.7328 |
| Caffeic acid | Time | 1 | 0.3188 | 0.0530 | 6.020 | 0.0209 | **0.0348** |
| Caffeic acid | Irradiation | 4 | 0.2361 | 0.0530 | 4.458 | 0.0068 | **0.0339** |
| Caffeic acid | Time × Irradiation | 4 | 0.0958 | 0.0530 | 1.809 | 0.1562 | 0.5161 |
| p-Coumaric acid | Time | 1 | 35.61 | 6.282 | 5.669 | 0.0246 | **0.0361** |
| p-Coumaric acid | Irradiation | 4 | 17.82 | 6.282 | 2.836 | 0.0438 | 0.1218 |
| p-Coumaric acid | Time × Irradiation | 4 | 5.700 | 6.282 | 0.907 | 0.4737 | 0.6579 |
| Ferulic acid | Time | 1 | 145.00 | 0.8640 | 167.82 | <0.0001 | **<0.0001** |
| Ferulic acid | Irradiation | 4 | 32.09 | 0.8640 | 37.137 | <0.0001 | **<0.0001** |
| Ferulic acid | Time × Irradiation | 4 | 1.273 | 0.8640 | 1.473 | 0.2379 | 0.5407 |
| trans-Resveratrol | Time | 1 | 0.2435 | 0.7804 | 0.312 | 0.5811 | 0.6053 |
| trans-Resveratrol | Irradiation | 4 | 2.295 | 0.7804 | 2.941 | 0.0386 | 0.1207 |
| trans-Resveratrol | Time × Irradiation | 4 | 1.365 | 0.7804 | 1.749 | 0.1685 | 0.5161 |
| (+)-Catechin | Time | 1 | 2791.0 | 128.00 | 21.805 | <0.0001 | **0.0004** |
| (+)-Catechin | Irradiation | 4 | 161.54 | 128.00 | 1.262 | 0.3091 | 0.4067 |
| (+)-Catechin | Time × Irradiation | 4 | 22.67 | 128.00 | 0.177 | 0.9482 | 0.9667 |
| (–)-Epicatechin | Time | 1 | 1094.0 | 21.97 | 49.785 | <0.0001 | **<0.0001** |
| (–)-Epicatechin | Irradiation | 4 | 31.53 | 21.97 | 1.435 | 0.2496 | 0.4067 |
| (–)-Epicatechin | Time × Irradiation | 4 | 22.29 | 21.97 | 1.014 | 0.4175 | 0.6140 |
| Epicatechin gallate | Time | 1 | 11.33 | 1.990 | 5.695 | 0.0243 | **0.0361** |
| Epicatechin gallate | Irradiation | 4 | 10.03 | 1.990 | 5.042 | 0.0036 | **0.0227** |
| Epicatechin gallate | Time × Irradiation | 4 | 3.121 | 1.990 | 1.568 | 0.2112 | 0.5280 |
| Quercetin 3-O-glucoside | Time | 1 | 2027.5 | 463.11 | 4.378 | 0.0459 | 0.0607 |
| Quercetin 3-O-glucoside | Irradiation | 4 | 623.04 | 463.11 | 1.345 | 0.2789 | 0.4067 |
| Quercetin 3-O-glucoside | Time × Irradiation | 4 | 195.09 | 463.11 | 0.421 | 0.7919 | 0.8607 |
| Quercetin 3-O-glucuronide | Time | 1 | 1272.3 | 385.77 | 3.298 | 0.0805 | 0.1006 |
| Quercetin 3-O-glucuronide | Irradiation | 4 | 971.65 | 385.77 | 2.519 | 0.0646 | 0.1614 |
| Quercetin 3-O-glucuronide | Time × Irradiation | 4 | 471.25 | 385.77 | 1.222 | 0.3249 | 0.5611 |
| Total coloured phenolics | Time | 1 | 2.071e+06 | 2.782e+05 | 7.444 | 0.0111 | **0.0202** |
| Total coloured phenolics | Irradiation | 4 | 3.295e+05 | 2.782e+05 | 1.184 | 0.3400 | 0.4250 |
| Total coloured phenolics | Time × Irradiation | 4 | 3.170e+05 | 2.782e+05 | 1.139 | 0.3591 | 0.5611 |
| Total non-coloured phenolics | Time | 1 | 44708.4 | 3321.0 | 13.462 | 0.0011 | **0.0029** |
| Total non-coloured phenolics | Irradiation | 4 | 7206.0 | 3321.0 | 2.170 | 0.0995 | 0.2260 |
| Total non-coloured phenolics | Time × Irradiation | 4 | 1855.5 | 3321.0 | 0.559 | 0.6945 | 0.7893 |

The model included storage time, irradiation dose, their interaction, and source bag as the blocking factor. Degrees of freedom were 1 for time, 4 for irradiation dose, 4 for the interaction, and 27 for the residual error. Raw p-values were adjusted using the Benjamini–Hochberg false discovery rate procedure separately for each model term across all estimable response variables within the pomace matrix. FDR-adjusted q-values < 0.05 are shown in bold. Kaempferol 3-O-glucoside and the quercetin derivative were not estimable in MAP because the corresponding values were constant or below the quantification limit.

**Fig S1.** Partial Least Squares Discriminant Analysis (PLS-DA) score plots for grape pomace extracts 1 day after gamma irradiation: (A) unfermented Sauvignon blanc pomace (SBP) and (B) fermented Malbec pomace (MAP). The models included phenolic compounds, mineral elements, and antioxidant-related variables and were fitted using two latent components. Model performance was evaluated using four-fold leave-one-block-out cross-validation. For SBP, R²X = 0.395, R²Y = 0.363, Q² = 0.037, and classification accuracy = 45%; for MAP, R²X = 0.463, R²Y = 0.362, Q² = 0.065, and classification accuracy = 55%.

**Fig S2.** Variable Importance in Projection (VIP) scores derived from the PLS-DA models for grape pomace extracts 1 day after gamma irradiation: (A) unfermented Sauvignon blanc pomace (SBP) and (B) fermented Malbec pomace (MAP). The analysis included phenolic compounds and mineral elements. The dashed line indicates the VIP threshold of 1; variables with VIP scores > 1 were considered candidate contributors to the observed treatment-related separation.

**Abbreviations used in the figures**

*UP, total quantified uncoloured phenolics; CP, total quantified coloured phenolics; K 3-O-glc, kaempferol 3-O-glucoside; Q 3-O-glc, quercetin 3-O-glucoside; Q 3-O-glcA, quercetin 3-O-glucuronide; Q derivative, quercetin derivative; Dp 3-O-glc, delphinidin 3-O-glucoside; Cy 3-O-glc, cyanidin 3-O-glucoside; Pt 3-O-glc, petunidin 3-O-glucoside; Mv 3-O-glc, malvidin 3-O-glucoside; Dp 3-acet, delphinidin 3-O-acetylglucoside; Pt 3-acet, petunidin 3-O-acetylglucoside; Pn 3-acet, peonidin 3-O-acetylglucoside; Mv 3-acet, malvidin 3-O-acetylglucoside; Cy 3-p-cou, cyanidin 3-O-p-coumaroylglucoside; Pt 3-p-cou, petunidin 3-O-p-coumaroylglucoside; Pn 3-p-cou, peonidin 3-O-p-coumaroylglucoside; Mv 3-p-cou, malvidin 3-O-p-coumaroylglucoside; DPPH, 2,2-diphenyl-1-picrylhydrazyl radical-scavenging capacity; ORAC, oxygen radical absorbance capacity; P, phosphorus; K, potassium; Ca, calcium; Mg, magnesium; Fe, iron; Mn, manganese; Zn, zinc; Cu, copper; B, boron.*
